# *Lactiplantibacillus plantarum* uses ecologically relevant, exogenous quinones for extracellular electron transfer

**DOI:** 10.1128/mbio.02234-23

**Published:** 2023-11-20

**Authors:** Eric T. Stevens, Wannes Van Beeck, Benjamin Blackburn, Sara Tejedor-Sanz, Alycia R. M. Rasmussen, Mackenzie E. Carter, Emily Mevers, Caroline M. Ajo-Franklin, Maria L. Marco

**Affiliations:** 1 Department of Food Science and Technology, University of California‐Davis, Davis, California, USA; 2 Department of Chemistry, Virginia Polytechnic Institute and State University, Blacksburg, Virginia, USA; 3 Biological Nanostructures Facility, The Molecular Foundry, Lawrence Berkeley National Laboratory, Berkeley, California, USA; 4 Department of Biosciences, Rice University, Houston, USA; The University of Queensland, Brisbane, Queensland, Australia

**Keywords:** lactic acid bacteria, cross-feeding, oxidation-reduction, transcriptome, fermentation

## Abstract

Extracellular electron transfer (EET) is a metabolic process that frequently uses quinones to couple intracellular redox reactions with extracellular electron acceptors. The physiological relevance of this metabolism for microorganisms capable of EET but unable to synthesize their own quinones remains to be determined. To address this question, we investigated quinone utilization by *Lactiplantibacillus plantarum*, a microorganism required for food fermentations, that performs EET and is also a quinone auxotroph. *L. plantarum* selectively used 1,4-dihydroxy-2-naphthoic acid (DHNA) and more hydrophilic naphthoquinones for EET reduction of insoluble iron (ferrihydrite). However, quinones used for EET also inhibited *L. plantarum* growth in non-aerated conditions. Transcriptomic analysis showed that DHNA-induced oxidative stress in *L. plantarum,* but this was alleviated when the electron acceptor, ferric ammonium citrate (FeAC), was included in the growth medium. Although DHNA and FeAC induced *L. plantarum* EET, this metabolism was still dependent on direct access to environmental electron shuttles. To determine whether quinone-producing food fermentation bacteria could be sources of those electron shuttles, *L. plantarum* EET was measured after incubation with *Lactococcus lactis* and *Leuconostoc mesenteroides*. Quinone-producing *L. lactis*, but not a quinone-deficient *L. lactis* Δ*menC* mutant, increased *L. plantarum* ferrihydrite reduction and medium acidification through an EET-dependent mechanism. *L. plantarum* EET was also stimulated by *L. mesenteroides*, resulting in greater environmental acidification and transient increases in *L. plantarum* cell numbers. Our findings show that *L. plantarum* overcomes the toxic effects of exogenous quinones to use those compounds for EET-conferred, ecological advantages during the early stages of food fermentations.

**IMPORTANCE:**

While quinones are essential for respiratory microorganisms, their importance for microbes that rely on fermentation metabolism is not understood. This gap in knowledge hinders our understanding of anaerobic microbial habitats, such in mammalian digestive tracts and fermented foods. We show that *Lactiplantibacillus plantarum,* a model fermentative lactic acid bacteria species abundant in human, animal, and insect microbiomes and fermented foods, uses multiple exogenous, environmental quinones as electron shuttles for a hybrid metabolism involving EET. Interestingly, quinones both stimulate this metabolism as well as cause oxidative stress when extracellular electron acceptors are absent. We also found that quinone-producing, lactic acid bacteria species commonly enriched together with *L. plantarum* in food fermentations accelerate *L. plantarum* growth and medium acidification through a mainly quinone- and EET-dependent mechanism. Thus, our work provides evidence of quinone cross-feeding as a key ecological feature of anaerobic microbial habitats.

## INTRODUCTION

Oxidation-reduction (redox) reactions dictate the flow of electrons in many enzymatic processes. Accordingly, these reactions are facilitated by an abundance of shuttling compounds used by bacteria to transport electrons within and outside of the cell (1). Quinones are one such class of diverse, redox-active molecules with over 1,000 structural forms (2). Quinones serve numerous functions in cells in all domains of life including in electron transport chains used for respiration energy conservation metabolism, extracellular protein folding, and pyrimidine synthesis (3). Conversely, these compounds can also cause cellular damage. Quinones reduce oxygen to reactive oxygen species (ROS) that result in DNA damage and membrane lipid peroxidation (4). Exogenous quinones can also inhibit respiration by competing with native quinones produced by the cell (5). The conflicting impacts of quinones on energy conservation pathways illustrate the complexity of their effects. Furthermore, their importance for microorganisms that do not rely on respiratory metabolism remains unclear.

Quinones are frequently utilized for extracellular electron transfer (EET), a metabolic process that couples intracellular redox reactions to extracellular electron acceptors or donors like redox-active metals or electrodes (6, 7). EET metabolism can be categorized based on the direction of electron flow to and from the electro-active microorganism and how electron transfer is completed (7, 8). The reduction of long-range electron acceptors through mediated electron transfer occurs with endogenous, membrane-associated quinones used as intracellular electron shuttles and with environmental or secreted quinones functioning as extracellular electron shuttles (9, 10). These EET pathways have thus far been best characterized for species of the Gram-negative, mineral-respiring bacteria *Shewanella* and *Geobacter* (11), although the number of EET active microorganisms is now understood to extend to all three domains of life (12).

We recently showed that *Lactiplantibacillus plantarum* performs EET by a blended metabolism combining features of respiration and fermentation (13). *L. plantarum* is a member of the lactic acid bacteria (LAB), a group of Gram-positive bacteria in the Bacillota (formerly Firmicutes) phylum that share metabolic and physiological characteristics and named for their production of lactic acid from fermentation energy conservation metabolism (14). *L. plantarum* EET partially resembles respiration because it increases intracellular NAD^+^:NADH ratios, but fermentation energy conversation metabolism with substrate-level phosphorylation is still used for ATP generation. *L. plantarum* EET results in a shortened lag phase, increased fermentation flux through organic acid production, and greater environmental acidification (13). The *L. plantarum* EET pathway is present in other LAB and similar to the flavin-mediated extracellular electron transfer (FLEET) system encoded by *Listeria monocytogenes*, a mainly respiratory Gram-positive species (9, 15). We found that *L. plantarum* NCIMB8826R requires *ndh2*, encoding a type II NADH dehydrogenase, and conditionally requires *pplA*, predicted to encode a flavin-binding membrane reductase, in the FLEET pathway to reduce extracellular ferric iron or a polarized anode (13, 16).

Despite the benefits of EET for *L. plantarum* growth and intracellular redox and energy homeostasis, members of this species lack the capacity to synthesize either flavins or quinones (17, 18). Whereas exogenous flavins are essential for *L. plantarum* growth under all conditions, quinones are only variably needed. EET reduction of insoluble ferrihydrite (iron[III] oxyhydroxide) and production of current in a bioelectrochemical reactor by *L. plantarum* NCIMB8826R are dependent on exogenous quinones (13). *L. plantarum* NCIMB8826R does contain several genes hypothesized to condense 1,4-dihydroxy-2-naphthoic acid (DHNA) with an isoprenoid polymer to form demethylmenaquinone (DMK), the membrane electron carrier used by *L. monocytogenes* (9) and *Enterococcus faecalis* (19) for EET. However, *L. plantarum* lacks the other genes required for a complete menaquinone biosynthesis pathway (20). Instead, exogenous DHNA, a quinone present in foods and made by other bacteria, was sufficient for *L. plantarum* EET metabolism (13, 16).

To better understand the sources and impacts of quinones on *L. plantarum* and its use of EET, we investigated the quinone diversity and concentration ranges used by *L. plantarum* for EET, the effects of those compounds on *L. plantarum* growth, and the significance of quinones produced by other LAB for *L. plantarum* in food fermentations. Our results reveal how *L. plantarum* EET metabolism is adapted for exogenous environmental quinones and that *L. plantarum* engages in quinone cross-feeding with other food fermentation bacteria. The findings have significance for understanding the ecological and physiological importance of EET in microorganisms that primarily rely on fermentation for energy conservation.

## RESULTS

### *L. plantarum* quinone selectivity for EET

**Fig 1:**
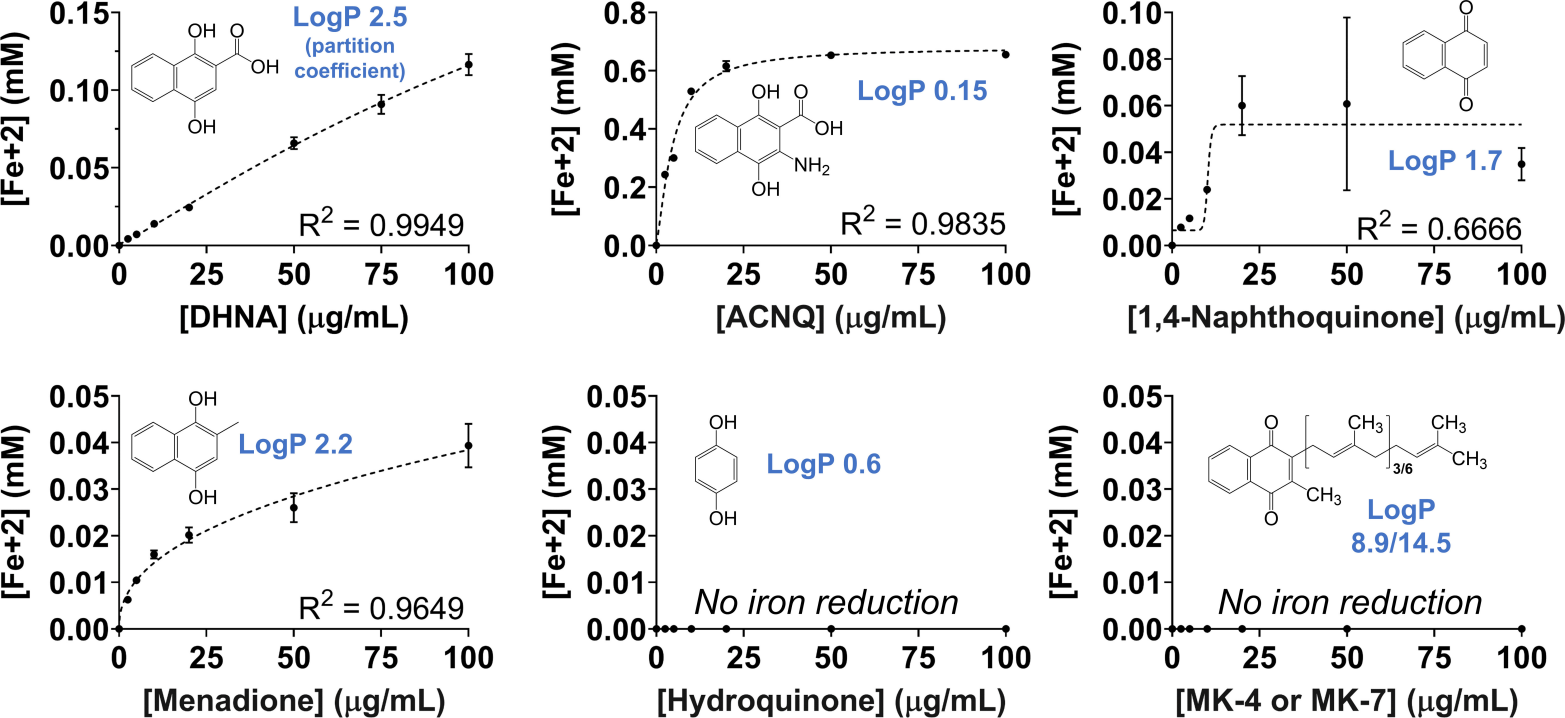
*L. plantarum* uses multiple quinone species for iron reduction. Ferrihydrite reduction assays were performed in phosphate-buffered saline containing 55 mM mannitol and quinones at the specified concentrations. *L. plantarum* cells used in the assay were grown in modified MRS medium with mannitol (mMRS) without quinone supplementation. The *R*^2^ value corresponds to the sigmoidal dose-response curve fit to the data. The chemical structure of each quinone and its corresponding log_P_ (partition coefficient) are provided. The average ± standard deviation of three biological replicates is shown.

We previously reported that EET reduction of insoluble ferrihydrite and production of current in a bioelectrochemical reactor by our model strain, *L. plantarum* NCIMB8826R, are dependent on the presence of exogenous DHNA in the assay medium (13). To determine whether this activity is specific to DHNA or if other ecologically relevant quinones can be used, ferrihydrite reduction was measured after *L. plantarum* growth and subsequent incubation in mannitol (55 mM) as an energy source and different concentrations of either DHNA, 2-amino-3-carboxy-1,4-naphthoquinone (ACNQ), menadione, hydroquinone, phylloquinone (phytomenadione, vitamin K1), 1,4-naphthoquinone, or the menaquinones MK-4 and MK-7. DHNA, ACNQ, and menadione are produced by bacteria (10, 21, 22). The quinone precursor hydroquinone is found in both animal and fungal tissues (23, 24). Phylloquinone and its precursor 1,4-naphthoquinone are contained in plant tissues (22). MK-4 and MK-7 are present in animal tissues and are also produced by bacteria (25). In addition to DHNA, *L. plantarum* reduced ferrihydrite when ACNQ, 1,4-naphthoquinone, or menadione were included in the assay medium (Fig. 1). ACNQ, a soluble analog of DHNA, resulted in the highest level of ferrihydrite reduction by *L. plantarum* with a maximum of 0.68-mM Fe^2+^ reduced, and a half maximal effective concentration (EIC_50_) of 4.70-µg/mL. *L. plantarum* also used 1,4-naphthoquinone (0.05 Fe^2+^ max, EIC_50_ = 10.23 µg/mL) and menadione (0.05 Fe^2+^ max, EIC_50_ = 29.79 µg/mL) to reduce iron, but less effectively than DHNA (0.40 Fe^2+^ max, EIC_50_ = 223.4 µg/mL). In contrast, ferrihydrite was not reduced by *L. plantarum* in the presence of hydroquinone, phylloquinone, MK-4, or MK-7 up to the maximum concentration tested (100 µg/mL) (Fig. 1). The high partitioning coefficients (log_P_) of those quinones suggest that *L. plantarum* uses naphthoquinone-based, hydrophilic quinones for EET. These findings show that *L. plantarum* quinone requirements for EET are not limited to DHNA and that other environmental quinones are sufficient, providing a range of selectivity.

### Quinones induce oxidative stress inhibiting *L. plantarum* growth

**Fig 2:**
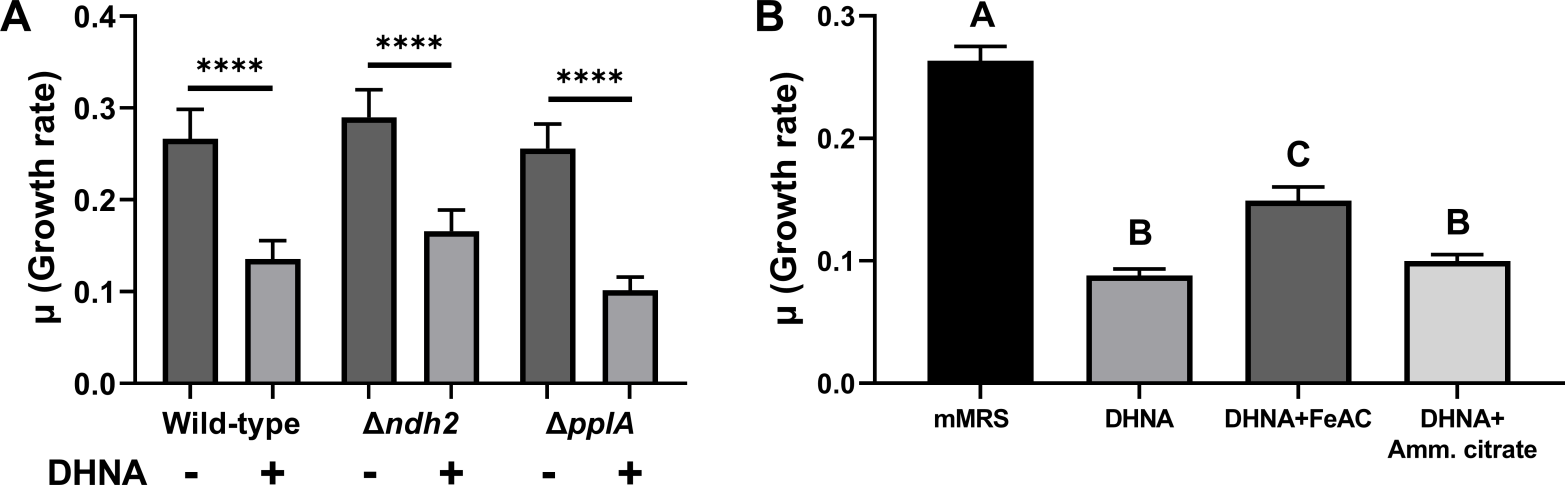
DHNA reduces *L. plantarum* growth, and this is ameliorated by soluble ferric iron. (**A**) Growth rate of wild-type *L. plantarum* NCIMB8826R and the Δ*ndh2* and *ΔpplA* mutants, MLES100 and MLES101, respectively, in mMRS with or without supplementation of 20-µg/mL DHNA. (**B**) Growth rate of *L. plantarum* NCIMB8826R in mMRS with or without supplementation of 20 µg/mL DHNA and 1.25 mM ferric ammonium citrate (FeAC) or 1.25 mM ammonium citrate. Growth rates were quantified by measuring the change in OD_600_ per hour during exponential phase. Significant differences represented by asterisks (****P ≤ 0.0001) and letters (*P* ≤ 0.05) were determined using one-way analysis of variance with Tukey’s post hoc test. The average ± standard error of the mean of three biological replicates is shown.

If *L. plantarum* uses EET as a metabolic strategy in food fermentations, its growth should not be negatively impacted by antimicrobial activity associated with exposure to quinones (4). Contrary to this assertion, we observed that under the static, non-aerated growth conditions common for food fermentations, *L. plantarum* was inhibited by DHNA and growth rates declined significantly when DHNA was added to the laboratory culture medium (Fig. 2; Fig. S1). In mMRS with 5-µg/mL DHNA, *L. plantarum* growth rates were reduced from 0.26 ± 0.01/h (mMRS) to 0.22 ± 0.01/h (*P* < 0.05) (Fig. S1). Growth rate decreased exponentially as a function of increasing DHNA concentrations, and no growth was observed at or above 150-µg/mL DHNA. This effect was not due to activation of EET because growth of the *L. plantarum* Δ*ndh2* and Δ*pplA* mutants was also negatively affected when DHNA was included in mMRS (Fig. 2A; Fig. S2). Similar effects on *L. plantarum* growth were found in mMRS containing other EET-conducive quinones (ACNQ, menadione, or 1,4-naphthoquinone) (Fig. S1). There was no observable increase in *L. plantarum* cell numbers when either menadione or 1,4-naphthoquinone was added at a concentration of 50 µg/mL. ACNQ was less toxic, but *L. plantarum* growth stopped at 100 µg/mL. Because ACNQ also conferred the highest EET activity, measured by quantities of iron reduced (Fig. 1), the reductions in *L. plantarum* growth were not directly related to the use of the quinone as an electron shuttle. These findings indicate that while *L. plantarum* can use different quinones as electron shuttles, exposure to these compounds could come at the cost of reducing overall fitness.

**Fig 3:**
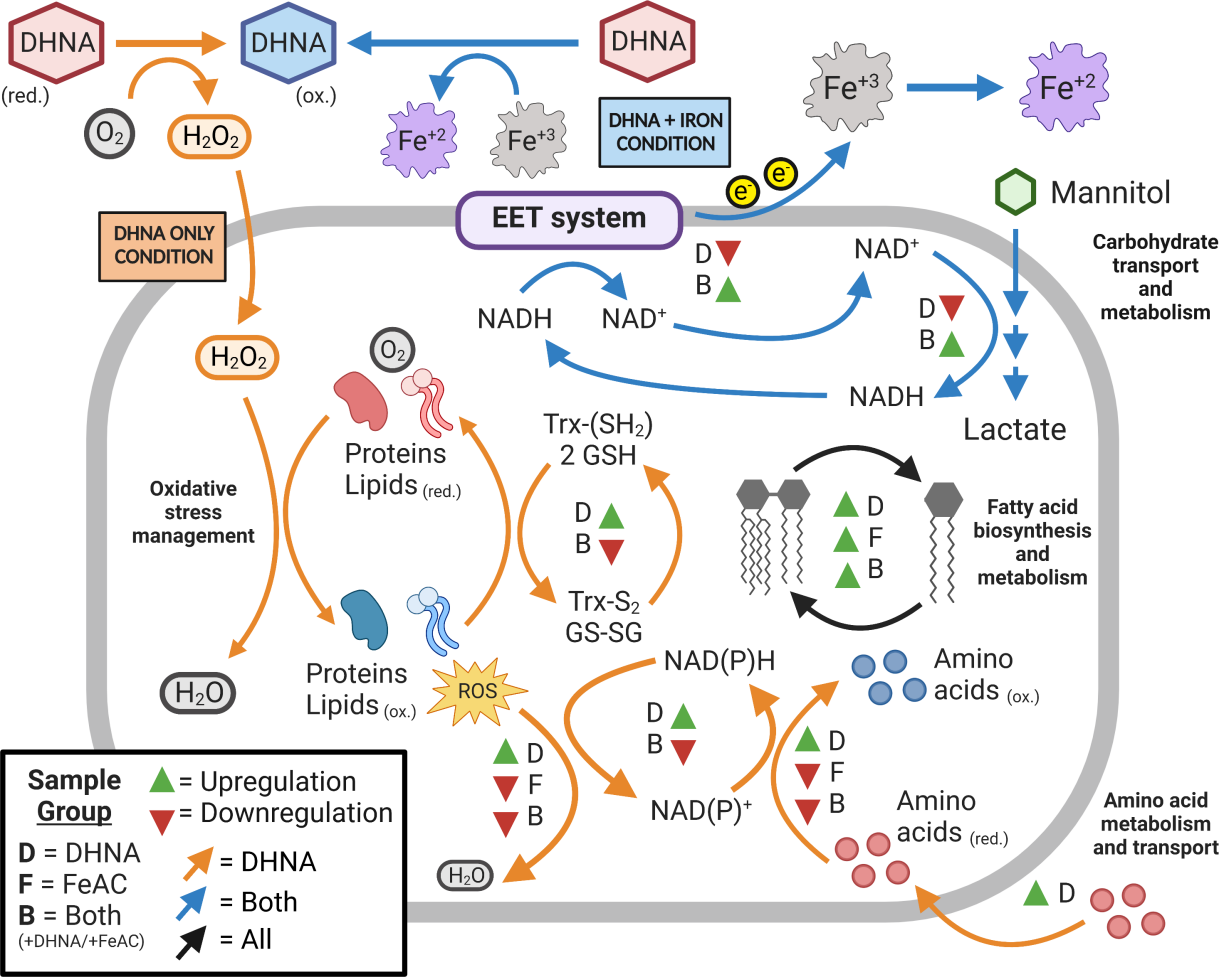
Induction of oxidative stress responses by DHNA is alleviated by FeAC. Summary of *L. plantarum* transcriptome changes in the presence of DHNA, FeAC, or DHNA + FeAC during exponential-phase growth in mMRS. Statistically significant changes in gene expression were determined using DEseq2 with a log_2_ expression fold change of ≥0.5 and false discovery rate-adjusted *P* value of ≤0.05. D, DHNA; F, FeAC; B, both DHNA + FeAC. Figure created with Biorender.com.

To understand why growth was reduced in the presence of quinones, we measured the transcriptomic responses of exponential-phase *L. plantarum* in mMRS supplemented with 20-µg/mL DHNA. Compared to cells in mMRS, expression levels of approximately 30% of the genes in the *L. plantarum* genome (452 genes upregulated and 473 genes downregulated) were affected (Table S1). Pathway analysis of the transcriptionally induced genes showed that *L. plantarum* was undergoing oxidative stress (Fig. 3; Tables S2 and S3). Transcripts for the genes encoding glutathione (*gshR2*/*R3*/*R4*), methionine-S-oxide reductase (*msrA2* and *msrB*), thioredoxin (*trxA2, trxA3*), and thioredoxin reductase (*trxB*) increased between 1.5-fold and 7.0-fold during incubation in DHNA. Likewise, genes for inactivating ROS including NADH oxidase (*nox5*), NADH peroxidase (*npr2*), catalase (*kat*), and pyruvate oxidase (*pox3*) were similarly upregulated. Transporters for sulfur-containing amino acids, as well as methionine and chorismate biosynthesis genes previously found to protect against oxidative stress (26–29), were also induced (Table S2). Lastly, the induction of membrane biosynthetic pathways (acetyl-CoA carboxylase [*accA2*, *accB2*, *accC2*, and *accD2*]) and fatty acid biosynthesis (*fabD*, *fabF*, *fabG*, *fabH2*, *fabI*, and *fabZ1*) were consistent responses to lipid peroxidation as observed for *Escherichia coli* (30).

Because the transcriptional responses of *L. plantarum* to DHNA signaled the activation of pathways responsive to intracellular oxidative stress, we measured the concentration of hydrogen peroxide (H_2_O_2_) in the mMRS medium. It was previously shown that H_2_O_2_ and other ROS are formed as a result of quinone hydroxyl groups reacting with O_2_ (31). Although *L. plantarum* cultures were not aerated, in order to resemble the normal growth conditions of LAB in food environments, a strict anaerobic environment was also not maintained. After 5-h incubation of *L. plantarum* in mMRS with DHNA, there was 29.92 ± 0.63 µM H_2_O_2_, a concentration approximately 15-fold higher than during *L. plantarum* growth in mMRS alone (Fig. S3). Sterile mMRS + DHNA contained even higher levels of that compound (41.90 ± 0.34 µM H_2_O_2_), indicating an abiotic interaction of mMRS with DHNA as opposed to H_2_O_2_ production by *L. plantarum*. Notably, however, the addition of catalase to DHNA-containing mMRS significantly reduced H_2_O_2_ concentrations but did not result in significant increases in *L. plantarum* growth rates (data not shown). Thus, the data show that inclusion of DHNA in the culture medium significantly increased the levels of H_2_O_2_, but the growth impairments were not due to the presence of that particular ROS alone.

### Electron acceptors alleviate DHNA-induced stress

Our initial findings revealed that including DHNA in laboratory culture medium together with soluble iron (ferric ammonium citrate [FeAC] [1.25 mM]) as an exogenous electron acceptor greatly improved EET in the post-growth ferrihydrite reduction assay (13). Consistent with FeAC increasing *L. plantarum* EET, the addition of this compound helped to alleviate some of the antimicrobial effects of DHNA and resulted in higher *L. plantarum* growth rates (0.15 ± 0.01/h) compared to when only DHNA was present (0.09 ± 0.01/h) (Fig. 2B; Fig. S4). The increase was due to the addition of iron and not citrate because there was no improvement in growth when ammonium citrate was added instead (Fig. 2B). Additionally, these effects of FeAC on *L. plantarum* were only observed when DHNA was also included in the mMRS. The growth rate of *L. plantarum* in the presence of FeAC was equivalent to the mMRS controls (Fig. S5). These data strongly suggest that iron reduces the oxidative stress generated by quinones in the presence of O_2_.

Consistent with higher growth rates of *L. plantarum* when FeAC was included together with DHNA in mMRS, the presence of FeAC resulted in lower H_2_O_2_ concentrations (8.34 ± 0.02 µM) (*P* < 0.0001) (Fig. S3). Similarly, *L. plantarum* genes required for oxidative stress tolerance and redox-associated amino acid metabolism were either unaffected or were downregulated during growth in mMRS with FeAC or mMRS with FeAC and DHNA (Fig. 3; Table S2). Transcriptional changes were not completely prevented with the addition of FeAC, however, and genes for lipid metabolism and cell membrane biosynthesis were upregulated (Table S2). These data establish that *L. plantarum* growth is inhibited by exogenous quinones, and the detrimental effects of DHNA are largely diminished when a terminal electron acceptor is present.

### Growth in DHNA and FeAC increases *L. plantarum* EET

**Fig 4:**
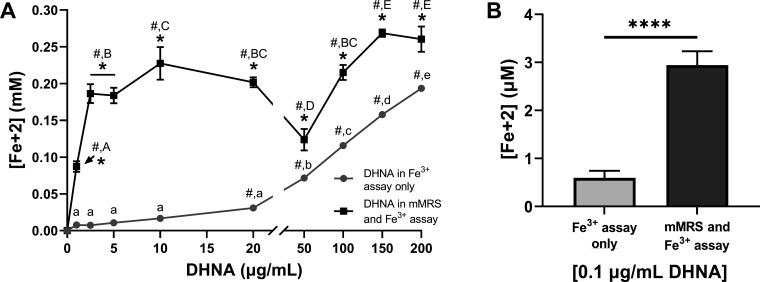
Growth in DHNA results in greater stimulation of *L. plantarum* EET. Reduction of Fe^3+^ (ferrihydrite) to Fe^2+^ by *L. plantarum* after growth in mMRS with 1.25 mM ferric ammonium citrate and DHNA. DHNA was supplemented in the growth medium and/or the ferrihydrite reduction assay at the indicated concentrations (**A**) or at 0.1 µg/mL (**B**). Significant differences were determined by one-way analysis of variance with Tukey’s post hoc test (*P* ≤ 0.05). Letters denote when iron reduction was significantly different between DHNA concentrations within the same growth condition. Asterisks indicate when iron reduction was significantly greater (*P* ≤ 0.05) when the same concentration of DHNA was either included or excluded from the mMRS growth medium. Pound signs denote when iron reduction was significantly greater (*P* ≤ 0.05) than when no DHNA was included in the ferrihydrite reduction assay. In panel B, ****P ≤ 0.0001. The average ± standard deviation of three biological replicates is shown.

Next, we investigated the extent that EET is enhanced when *L. plantarum* is grown in the presence of DHNA and FeAC and why this occurs. Significantly more iron was reduced in the post-growth ferrihydrite assay when these compounds were included in the mMRS laboratory culture medium (Fig. 4A and B). The effect of DHNA and FeAC was observed over a broad range of concentrations (0.1–200.0 µg/mL), and remarkably, the impact of adding it to mMRS was greatest when lower quantities of DHNA (<20 µg/mL) were used (Fig. 4A). Increased iron reduction was still observable when only 0.1 µg/mL DHNA was included (Fig. 4B). The findings therefore indicate that *L. plantarum* exposure to FeAC and DHNA during growth induces physiological changes that dramatically enhance its capacity to perform EET.

One possible response that could improve the capacity of *L. plantarum* to perform EET is the upregulation of genes within the FLEET locus. Growth of *L. plantarum* mMRS with DHNA and FeAC was previously observed to result in the induction of *ndh2* and *pplA* in the FLEET pathway (13) (Table S2). However, other genes in the FLEET locus were not induced, potentially indicating that they are dispensable for EET (Tables S2 and S3). In this regard, another study found that *L. plantarum* mutants lacking the FLEET locus genes encoding membrane DMK synthesis proteins (DmkA or EetB/DmkB) or the electron transfer protein EetA were still able to perform DHNA-dependent EET, albeit at a somewhat reduced level compared to the wild-type strain (16).

Instead, genes needed for anaerobic respiration, namely, those coding for nitrate reductase (*narGHJI*) and the molybdopterin cofactor (*moeA* and *moeB*), were induced in mMRS with DHNA and FeAC (Table S2). While nitrate reductase is not required for the reduction of extracellular electron acceptors (outward EET) (13), the *narGHJI* operon was implicated in electron uptake from an electrode by *L. plantarum* in a biochemical reactor to result a metabolic shift toward ATP-generating pathways and prolonged survival after sugar exhaustion (32). Therefore, higher quantities of Ndh2, PplA, and nitrate reductase in *L. plantarum* after growth in mMRS containing DHNA and FeAC likely provided an increased EET capacity and an improved energetic state in the post-growth ferrihydrite reduction assay.

### *L. plantarum* contains MK-6 and MK-7 after growth in DHNA but still relies on direct access to exogenous electron shuttles for EET

Transcriptomic analysis showed that genes required for menaquinone biosynthesis from DHNA, including *dmkA* (lp_1546), lp_1135, lp_1715, and *ubiE*, were not differentially expressed in mMRS containing either DHNA or DHNA and FeAC (Table S2). To determine whether DHNA could still be incorporated into *L. plantarum* cells irrespective of gene expression changes, intracellular quinones were measured by liquid chromatography-mass spectrometry (LCMS). After growth in mMRS with DHNA (with and without FeAC), menaquinone-6 (MK-6) and MK-7 were the only quinones detected and present in an approximate ratio of 13 (8.67 × 10^6^ ± 6.53 × 10^5^ MK-6 ion counts) to 1 (6.53 × 10^5^ ± 3.56 × 10^5^ MK-7 ion counts), respectively. No quinones were detected when DHNA was omitted from the culture medium (data not shown). Because similar quantities of MK-6 and MK-7 were recovered from *L. plantarum* after growth in EET-conducive (mMRS with DHNA and FeAC) and EET non-conducive conditions (mMRS with DHNA), it is expected that other factors besides the presence of intracellular quinones must be also involved in the heightened EET activity observed for *L. plantarum* incubated in mMRS with DHNA and FeAC.

**Fig 5:**
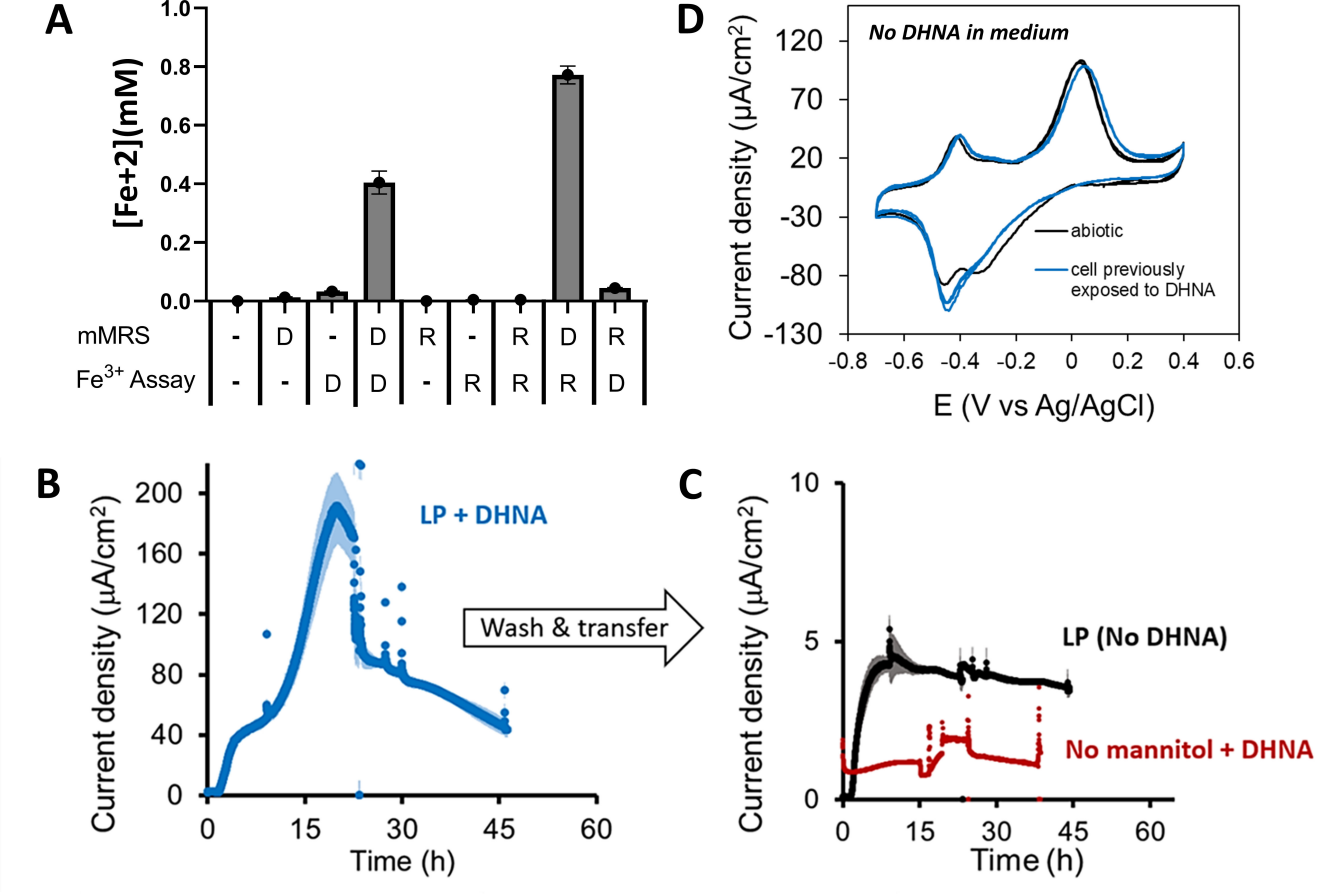
Access to exogenous electron shuttles is required for *L. plantarum* EET. (**A**) Ferrihydrite reduction measured after growth in mMRS with DHNA (D) or supplemental riboflavin (R) and variably exposed to these compounds in the post-growth ferrihydrite (Fe^3+^) assay. (**B**). Current density production over time by *L. plantarum* in chemically defined minimal medium with mannitol (mCDM) supplemented with 20-µg/mL DHNA. The anode was polarized at +0.2V Ag/AgCl. (**C**) Cells from panel **B** were washed and transferred to bioreactors containing mCDM with no DHNA or chemically defined minimal medium (CDM) with no mannitol or DHNA. (**D**) Cyclic voltammetry analysis of *L. plantarum* in mCDM with either prior growth in mCDM with 20-µg/mL DHNA or mCDM alone. The average + standard deviation of three biological replicates is shown.

Another physiological change that could increase EET metabolism is the secretion of quinones or other electron carriers into the extracellular environment. However, *L. plantarum* remained dependent on the addition of exogenous electron shuttles during the post-growth, ferrihydrite reduction assay (Fig. 5A). These shuttles are not limited to DHNA because riboflavin was also used by *L. plantarum* to reduce ferrihydrite (Fig. 5A). Moreover, in a bioelectrochemical reactor, *L. plantarum* generated current when DHNA was present, but this activity stopped when it was transferred to an electrochemical cell lacking an exogenous quinone source (Fig. 5B and C). The lack of secreted soluble redox-active mediators was confirmed by cyclic voltammetry (Fig. 5D), thus verifying that *L. plantarum* growth in the presence of DHNA and FeAC does not increase EET from the secretion of electron shuttles.

In summary, *L. plantarum* EET is inducible and more sensitive to low quantities of exogenous electron shuttles when *L. plantarum* is grown in the presence of DHNA and iron. *L. plantarum* harbors MK-6 and MK-7 when DHNA is present in the growth medium, but EET metabolism still requires additional exogenous electron shuttles such as quinones or flavins, along with an environment containing electron acceptors like soluble iron (FeAC). Thus, although the roles of MK-6 and MK-7 in *L. plantarum* and extracellular signals needed for increased expression of *ndh2* and *pplA* are not yet known, increases in EET may be stimulated by an elevated energetic state, the availability of Ndh2 and PplA required for ferrihydrite reduction, reduced oxidative stress, and the presence of an intracellular quinone pool.

### *L. plantarum* uses quinones made by other LAB for EET

**Fig 6:**
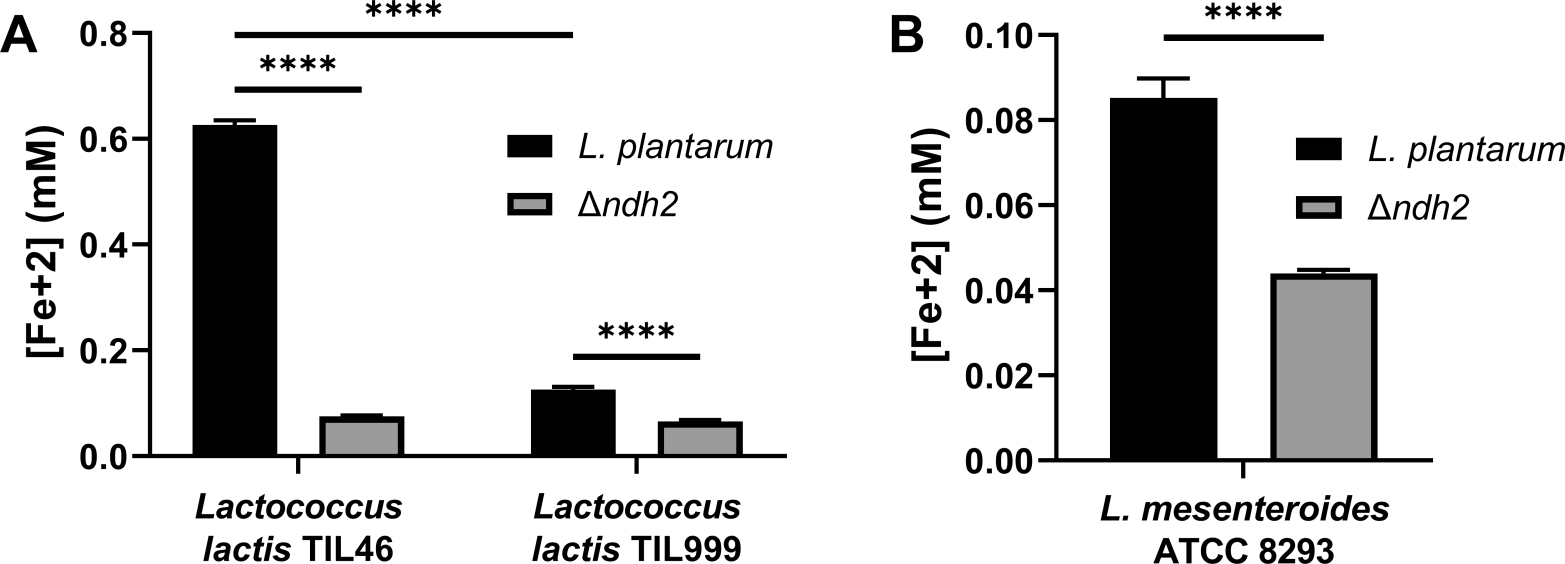
*L. plantarum* EET is activated by secreted compounds made by *L. lactis* and *Leuconostoc mesenteroides* in an EET-dependent manner. Ferrihydrite reduction by wild-type *L. plantarum* and the *ndh2* deletion mutant MLES100 in cell-free supernatant (CFS) from (**A**) wild-type *L. lactis* TIL46 and the *menC* deletion mutant TIL999 or (**B**) *L. mesenteroides* ATCC8293. Significant differences between groups (****P ≤ 0.0001) were determined by one-way analysis of variance with Tukey’s post hoc test. The average ± standard deviation of three biological replicates is shown.

In food fermentations, *L. plantarum* is frequently found together with LAB such as *Lactococcus lactis* and *Leuconostoc* spp., known to synthesize quinones (10, 33, 34). To determine whether the secreted products from those LAB influence *L. plantarum* EET activity, we modified the ferrihydrite reduction assay (Fig. S6A) to use cell-free, spent-medium (CFS) instead of purified quinones as electron shuttles. This assay showed that CFS collected after growth of *Lactococcus lactis* TIL46 contained compounds that enabled *L. plantarum-*mediated reduction of ferrihydrite (Fig. 6A). By comparison, minimal levels of insoluble iron were reduced upon *L. plantarum* incubation in CFS from TIL999, a quinone-deficient, *menC* deletion mutant of TIL46 (35) (Fig. 6A). These findings are consistent with *L. lactis* intracellular quinone concentrations, because whereas TIL46 cells contained MK-7, MK-8, and MK-9 in a ratio of 1:7:9, menaquinone quantities were significantly reduced in TIL999 to approximately 16-fold lower levels (Table 1). The lack of ferrihydrite reduction when *L. plantarum* Δ*ndh2* was incubated with the CFS from either *L. lactis* strain confirmed that the increase in activity was also dependent on an intact *L. plantarum* FLEET pathway (Fig. 6A).

**TABLE 1:** Cellular menaquinones in lactic acid bacteria^*a*^

Strain	Menaquinone normalized to cell pellet					
	MK6	MK7	MK8	MK9	MK10	MK11
*L. plantarum* NCIMB8826R	107.28 ± 23.07	11.29 ± 7.51	ND	ND	ND	ND
*L. lactis* TIL46	151.09 ± 32.87	471.12 ± 172.65	3,265.06 ± 1,244.88	4,297.39 ± 1,904.09	261.46 ± 146.37	ND
*L. lactis* TIL999	8.92 ± 5.15	ND	8.90 ± 2.85	109.60 ± 118.46	ND	ND
*L. mesenteroides* ATCC8293	ND	ND	0.08 ± 0.15	4.28 ± 2.38	124.94 ± 34.87	312.42 ± 97.64

^*a*^ Mean peak area per gram cell pellet ± standard deviation (*n* = 3) is provided*.* ND, none detected.

To assess whether *L. plantarum* EET stimulation is limited to *L. lactis* CFS, we also examined CFS from *Leuconostoc mesenteroides* ATCC8293. This strain was confirmed to produce quinones, and both MK-10 and MK-11 were present and detected in a ratio of 1:3, respectively (Table 1). Although the intracellular quinones in *L. mesenteroides* ATCC8293 differed from TIL46, the CFS from ATCC8293 also enabled *L. plantarum* ferrihydrite reduction (Fig. 6B). This activity was dependent on the presence of *L. plantarum ndh2* (Fig. 6B). In summary, these results strongly indicate that quinones synthesized by LAB reach the extracellular environment and can be used by *L. plantarum* as electron shuttles for EET reduction of insoluble iron.

Because the CFS from quinone-producing LAB was sufficient for *L. plantarum* EET, we hypothesized that quinone cross-feeding in co-culture would stimulate *L. plantarum* iron reduction *in situ. L. plantarum* was incubated in equal proportions with either *L. lactis* TIL46, *L. lactis* TIL999, or *L. mesenteroides* ATCC8293 in a chemically defined minimal medium with glucose (gCDM) and 1.25-mM FeAC. The addition of FeAC into that medium also provided a terminal electron acceptor that we could use to measure EET *in situ* via colorimetric analysis (Fig. S6B). Although there was spontaneous reduction of FeAC in acidified gCDM (Fig. S7), this background level of iron reduction was very low (<0.07 mM Fe^2+^) for the pH ranges measured here.

**Fig 7:**
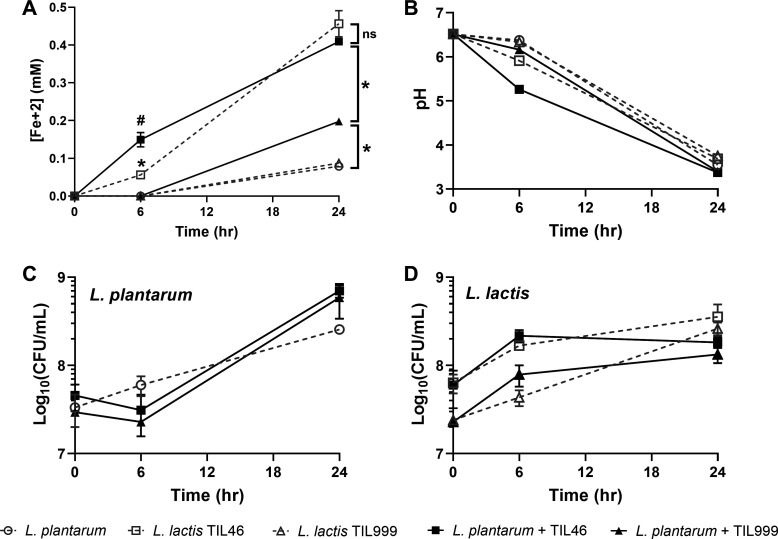
Co-culturing *L. plantarum* with quinone-producing *L. lactis* increases EET, acidification, and *L. plantarum* growth. (**A**) Ferrihydrite reduction by *L. plantarum* in gCDM with 1.25-mM FeAC during growth alone or in co-culture with *L. lactis* TIL49 or TIL999. Change in (**B**) pH and (**C**) *L. plantarum* and (**D**) *L. lactis* abundance over time when grown separately or combined in co-culture. Strains were grown in gCDM supplemented with 1.25-mM FeAC. Significant differences in ferrihydrite reduction of *L. plantarum* + TIL46 (#) and *L. lactis* TIL46 (*) compared to all other cultures at 6 h (P ≤ 0.05) and between all groups at 24 h (*P ≤ 0.05) were determined by two-way analysis of variance with Tukey’s post-hoc test. The average ± standard deviation of three biological replicates is shown.

After incubation of *L. plantarum* and *L. lactis* TIL46 in co-culture for 6 h, significantly more iron (0.149 ± 0.19 mM Fe^2+^) was reduced than when either *L. lactis* TIL46 (0.056 ± 0.01 mM Fe^2+^) or *L. plantarum* (no iron reduction) was grown separately (Fig. 7A). No iron reduction at this time point was found following TIL999 growth or the co-cultures of *L. plantarum* and TIL999 (Fig. 7A). By 24 h, *L. lactis* TIL46 and the co-cultures of *L. plantarum* and *L. lactis* TIL46 reduced similar amounts of iron (0.46 ± 0.04 mM Fe^2+^ and 0.41 ± 0.00 mM Fe^2+^, respectively) (Fig. 7A). These quantities were significantly greater than those found for either *L. plantarum* (0.079 ± 0.00 mM Fe^2+^) or TIL999 grown alone (0.087 ± 0.00 mM Fe^2+^). Although FeAC was reduced in the co-culture of *L. plantarum* and TIL999 (0.197 ± 0.00 mM Fe^2+^) at 24 h, this was significantly lower compared to cultures containing the *L. lactis* wild-type strain (Fig. 7A).

**Fig 8:**
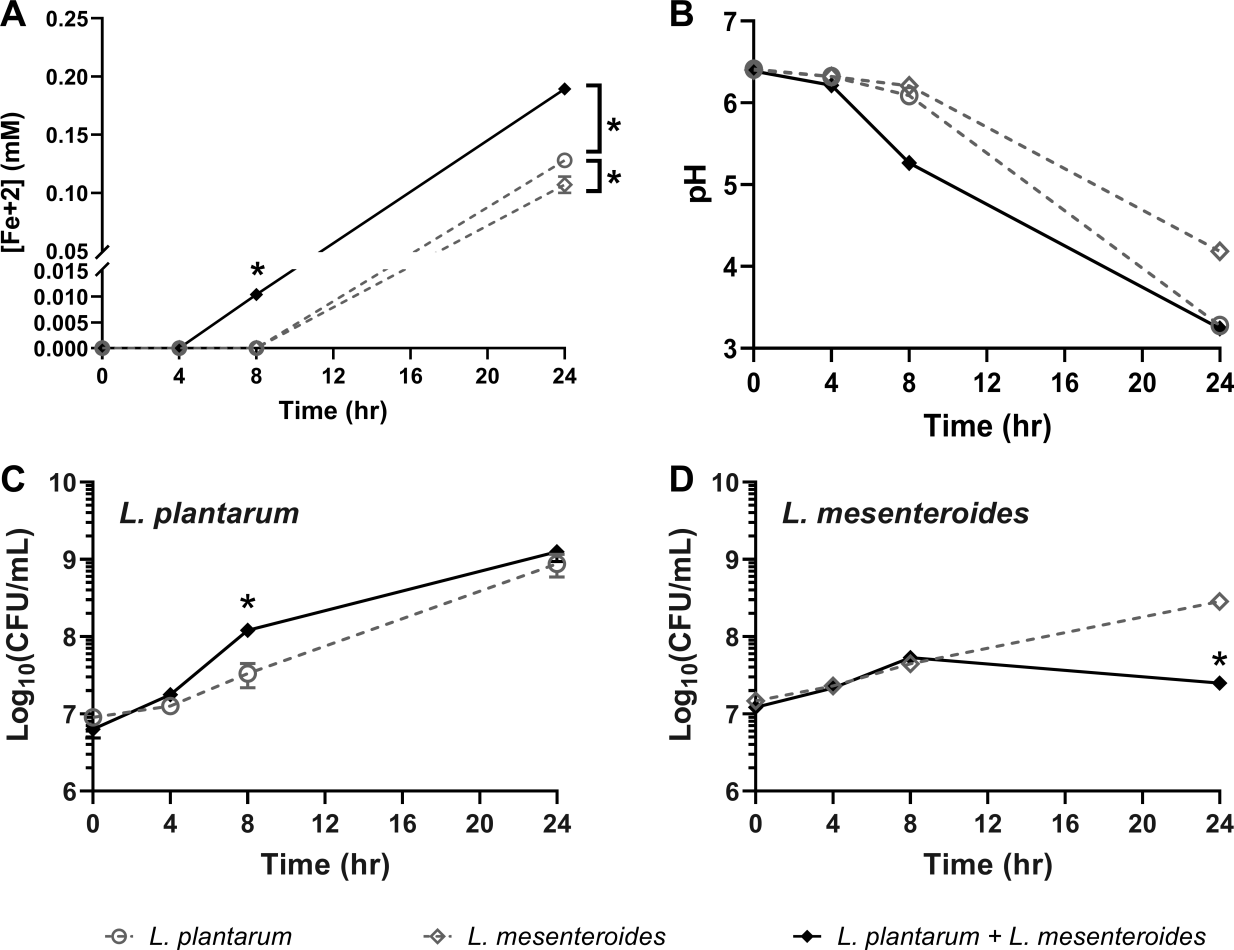
Co-culturing *L. plantarum* with quinone-producing *L. mesenteroides* increases EET, acidification, and *L. plantarum* growth. (**A**) Ferrihydrite reduction by *L. plantarum* in gCDM with 1.25 mM FeAC during growth alone or in co-culture with *L. mesenteroides* ATCC8293. Change in (**B**) pH and (**C**) *L. plantarum* and (**D**) *L. mesenteroides* abundance over time when grown separately or combined in co-culture. Strains were grown in gCDM supplemented with 1.25 mM FeAC. Significant differences in iron reduction or strain abundance were determined by two-way analysis of variance with Tukey’s post hoc test (*P ≤ 0.05) The average ± standard deviation of three biological replicates is shown.

For the co-cultures of *L. plantarum* and *L. mesenteroides*, FeAC reduction was found after incubation for 8 h (0.01 ± 0.00 mM Fe^2+^), whereas no iron was reduced by either strain grown separately (Fig. 8A). After 24 h, this value increased in the co-culture (0.19 ± 0.00 mM Fe^2+^) and remained significantly higher than the *L. plantarum* (0.13 ± 0.00 mM Fe^2+^) or *L. mesenteroides* (0.11 ± 0.01 mM Fe^2+^) cultures (Fig. 8A). These results show that environmental quinones secreted by other LAB can stimulate *L. plantarum* EET metabolism and that full quinone biosynthetic capabilities are necessary for robust iron reduction by *L. lactis*.

### Co-cultures of *L. plantarum* and quinone-producing LAB result in increased environmental acidification and altered growth

*L. plantarum* EET is associated with a shorter lag phase, increased viable cell counts, and accelerated environmental acidification (13). Because quinone-producing LAB promoted *L. plantarum* EET metabolism (Fig. 7A and 8A), we also quantified environmental acidification and strain growth after 6-h (*L. lactis*), 4- and 8-h (*L. mesenteroides*), and 24-h (*L. lactis* and *L. mesenteroides*) incubation of the strains individually and in co-culture with *L. plantarum*. For the *L. lactis* co-cultures, medium acidification was greater after 6 h when *L. plantarum* and *L. lactis* TIL46 were grown together (pH = 5.26 ± 0.03) than for the combination of *L. plantarum* and TIL999 (pH = 6.16 ± 0.03), as well as for *L. plantarum* (pH = 6.40 ± 0.01), TIL46 (pH = 5.91 ± 0.05), and TIL999 (pH = 6.34 ± 0.02) grown separately (Fig. 7B). Likewise, co-cultures of *L. plantarum* and *L. mesenteroides* ATCC8293 led to greater acidification of the medium (pH = 5.27 ± 0.05) within 8 h, compared to when the strains were grown separately (pH = 6.10 ± 0.01 and pH = 6.21 ± 0.01 for *L. plantarum* and *L. mesenteroides*, respectively) (Fig. 8B). Differences between co-culture pH values were no longer found after 24-h incubation (Figure 7B and 8B), consistent with previous observations that *L. plantarum* EET is active during the early exponential phase of growth (13). These data show that accelerated environmental acidification occurs when *L. plantarum* is grown together with quinone-producing LAB.

Besides the increased acidification rate, strain growth was altered in the *L. lactis* and *L. mesenteroides* co-cultures. When *L. lactis* was present, *L. plantarum* cell numbers remained static for the first 6 h and then were 2-fold higher after incubation for 24 h (Fig. 7C). However, this increase was not significant nor was it dependent on menaquinone biosynthetic capacity because both TIL46 and TIL999 resulted in similar increases in *L. plantarum* cell quantities (Fig. 7C). Growth of *L. lactis* was also not affected by *L. plantarum* (Fig. 7D). Conversely, the numbers of *L. plantarum* increased 4-fold upon 8-h incubation with *L. mesenteroides* compared to when it was grown separately (*P* = 0.03) (Fig. 8C). Growth of *L. mesenteroides* was negatively affected by co-culture, and 10-fold lower cell numbers were reached after 24-h incubation with *L. plantarum* (*P* = 0.04) (Fig. 8D). Hence, *L. plantarum* has antagonistic, inhibitory effects on *L. mesenteroides*, potentially resulting from the more rapid decline in medium pH.

## DISCUSSION

Quinones are abundant in fermented foods, human and animal microbiomes, as well as other environments, where they may be responsible for important ecological and physiological processes (3, 25, 36). We found that *L. plantarum,* a quinone auxotroph, can use multiple quinone species, including quinones made by other related bacteria, for EET. When an electron acceptor is present in food fermentation-relevant conditions, *L. plantarum* partially overcomes the growth inhibiting effects of DHNA. *L. plantarum* incorporates quinones into its intracellular contents when DHNA is present but still requires direct access to those compounds or other electron shuttles for EET. Increased initial environmental acidification during co-culture with quinone-producing *L. lactis* and *L. mesenteroides* and changes to *L. plantarum* and *L. mesenteroides* growth show the potential ecological significance of quinone-mediated EET for these mainly fermentative bacteria.

### *L. plantarum* uses hydrophilic exogenous quinones for EET

*L. plantarum* uses hydrophilic, naphthoquinone-based quinones with electron-withdrawing substituents such as menadione, ACNQ, and DHNA for EET. Many extracellular electron shuttles are small, hydrophilic molecules (37), and the presence of electron-withdrawing groups on quinones can stabilize radicals in the aromatic ring (38). EET activity increased logarithmically with quinone concentrations. For DHNA, the range of quinone use is comparable to concentrations found in makgeolli, a fermented Korean rice wine (0.089 to 0.44 µg/mL) (39) and produced by dairy-associated bacteria such as *Propionibacterium freudenreichii* (36.75 µg/mL) (40) and *Lacticaseibacillus casei* (0.37 µg/mL). Less is known about the quantities of menadione and ACNQ in *L. plantarum*-associated environments. However, *L. lactis* was previously shown to synthesize ACNQ (~43 µg/mL) (10, 41), and *Shewanella oneidensis* was found to produce up to 0.33-µg/mL ACNQ (10), which is consistent with the *L. plantarum* usage range of this quinone. In other LAB, 138-µg/mL menadione stimulated EET activity in *E. faecalis* (42). Likewise, approximately 2-µg/mL DHNA or ACNQ and approximately 14-µg/mL menadione were sufficient to stimulate EET in a *L. lactis menB* deletion mutant lacking DHNA biosynthesis capability (43). These data are also supported by our previous findings showing that ferrihydrite reduction occurred when 20-µg/mL DHNA was provided to several other LAB quinone auxotrophs, namely, *Lactiplantibacillus pentosus*, *L. casei*, and *Lacticaseibacillus rhamnosus* (13, 44).

*L. plantarum* EET was not observed in the presence of long-chain, hydrophobic menaquinones or phylloquinone. This differs from results showing that *S. oneidensis* can use MK-4 as well as ACNQ, DHNA, and menadione for EET at milligram concentrations (10). Notably, the CFS from *L. lactis* TIL46 and *L. mesenteroides* supported *L. plantarum* EET in a quinone-dependent manner, despite the finding that the intracellular contents of those LAB contained the long-chain menaquinones MK-7 through MK-11. While it is possible that some of those MKs could be used by *L. plantarum* for EET, it is also likely that MK intermediates or derivatives were released from *L. lactis* or *L. mesenteroides* during growth and were not measured by the cell-based LCMS performed here. To this regard, extracellular ACNQ was found in spent media from *L. lactis* (10, 43). Other LAB genera including *Leuconostoc*, *Weissella*, and *Enterococcus* are also capable of synthesizing DHNA (33) or menaquinones (44).

### Quinones induce oxidative stress, inhibiting *L. plantarum* growth, but this is alleviated by the addition of soluble iron

DHNA, ACNQ, menadione, and 1,4-naphthoquinone inhibited *L. plantarum* growth. While evidence of quinone-induced growth defects on LAB is limited, a recent study found that anaerobic growth of an *L. lactis* mutant incapable of DHNA biosynthesis was rescued with DHNA, ACNQ, or menadione supplementation (43). As with our results though, increasing concentrations of these three quinones resulted in decreased final cell densities (43). DHNA, menadione, and 1,4-naphthoquinone were also previously shown to confer antimicrobial activity against pathogens such as *Helicobacter pylori*, *Staphylococcus aureus*, and *Bacillus anthracis* (5, 45).

*L. plantarum* growth inhibition in the presence of the quinones was likely due to oxidative stress (31). *L. plantarum* relies on superoxide dismutase and intracellular H_2_O_2_-detoxification enzymes like NADH peroxidase, glutathione reductase, and thioredoxin reductase to prevent oxidative stress (46, 47). Many of these genes were upregulated in *L. plantarum* NCIMB8826R grown in mMRS with DHNA. A similar oxidative stress response was previously observed for *L. plantarum* CAUH2 upon exposure to 5 mM H_2_O_2_ (48). This finding suggests that *L. plantarum* activates similar stress-response pathways in the presence of quinones, compounds known to have complex cytotoxic effects (4). The upregulation of sulfur-containing amino acid transporters during growth in mMRS with DHNA is also consistent with an oxidative stress response. Both methionine and cysteine are radical-scavenging amino acids (49). Cysteine is incorporated into glutathione (26), and methionine is incorporated in S-adenosylmethionine for cystathionine production (50). Moreover, the induction of *L. plantarum* genes required for chorismate biosynthesis, a precursor compound to the membrane-associated antioxidant ubiquinol (27), further indicates a protective response to oxidative conditions present in mMRS with DHNA.

The growth rate of *L. plantarum* was significantly improved, and the concentration of extracellular hydrogen peroxide was reduced when FeAC was included with DHNA in the laboratory culture medium. Most oxidative stress responses were downregulated when FeAC was present. The presence of FeAC alone had minor effects on *L. plantarum* growth and gene expression. Even though *L. plantarum* can accumulate approximately 100 times higher quantities of iron during growth in FeAC (13), expression levels of *sufB* and *sufC* encoding iron-sulfur cluster assembly proteins used for detoxifying ROS in other LAB species (51) were downregulated in the FeAC-containing medium. This result is surprising because ferric iron was shown to catalyze ROS production in the presence of quinones (52). In mMRS, the complexing of iron with DHNA may make both compounds less available to react with molecular oxygen for ROS production (53). Thus, the addition of the terminal electron acceptor FeAC provided a means to reduce oxidative stress by lowering ROS synthesis.

### *L. plantarum* cells acquire quinones but still need access to environmental electron shuttles for EET

We found that although *L. plantarum* is a quinone auxotroph, it was still able to reduce extracellular ferrihydrite when incubated with DHNA and other hydrophilic quinones in the post-growth assay, and without prior inclusion of DHNA in the culture medium (Fig. 1 and 5A). Because membrane-bound quinones are thus far known to be essential for EET in many bacteria (54), it is possible that some quinone uptake occurred during that time. However, 5-fold higher levels of ferrihydrite were reduced when *L. plantarum* was first grown in laboratory culture medium containing DHNA and FeAC. That medium resulted in the induction of *ndh2* and *pplA* and the capacity of *L. plantarum* to use either DHNA or riboflavin as electron shuttles to reduce ferrihydrite. This increased EET capacity is likely due at least in part to the uptake and conversion of the exogenous quinone into its cellular contents. Because *L. plantarum* is able to respire using MK-4 for electron transport (17), these bacteria are apparently able to acquire different quinone species for intracellular electron transfer. Similarly, *L. monocytogenes* mutants unable to make either menaquinone or DMK lost the capacity for either aerobic respiration or EET metabolism, respectively (9).

*L. plantarum* intracellular quinones, encompassing the long-chain menaquinones MK-6 and MK-7, cannot be used by *L. plantarum* as extracellular electron shuttles for EET. These quinones also do not appear to be subsequently secreted because no soluble mediator was found in the culture medium after *L. plantarum* growth according to cyclic voltammetry or upon transfer of the cells from medium containing DHNA to another lacking quinones. This is unlike other bacteria such as *Enterobacter cloacae*, an organism that self-secretes hydroquinones as EET electron shuttles (55). Although the pathway for quinone incorporation into *L. plantarum* cells remains to be identified, it is notable that *L. plantarum* genes annotated for quinone metabolism were not induced when DHNA was included in the mMRS medium. While it is possible that the genes for menaquinone biosynthesis are constitutively expressed, it was also recently found that *L. plantarum* mutants lacking *dmkA* or *dmkB,* encoding a 1,4-dihydroxy-2-naphthoate polyprenyltransferase and heptaprenyl diphosphate synthase, respectively, were still able to perform EET in the presence of exogenous quinone (16). Therefore, it is expected that other, yet to be identified, pathways are used by *L. plantarum* for quinone uptake and modification.

### Quinone cross-feeding to *L. plantarum* has important ecological implications

Insoluble ferrihydrite was reduced during *L. plantarum* incubation in spent media (CFS) from either *L. lactis* TIL46 or *L. mesenteroides* ATCC8293, but not the quinone-deficient *L. lactis* TIL999 mutant, thus showing that *L. plantarum* EET was possible using electron shuttles made by other LAB strains. Although both *L. lactis* and *L. mesenteroides* are also capable of producing endogenous flavins (18) that can be utilized as environmental electron shuttles (16), the capacity of *L. lactis* to synthesize quinones was required for *L. plantarum* EET stimulation.

Notably, *L. lactis* spp., and to a lesser extent *L. mesenteroides*, were able to reduce FeAC on their own. Neither *L. lactis* nor *L. mesenteroides* possess a complete FLEET pathway (13). In addition, although *L. lactis* could generate an electric current (13) and reduce extracellular ferricyanide (43), it was unable to reduce ferrihydrite in the post-growth assay applied here (13). The observation that modest EET activity was observed with TIL999 shows there are other electron shuttles (e.g., flavins) generated by *L. lactis* that can be used by *L. plantarum* for EET, albeit with reduced efficiency. These findings reinforce the possibility that there are multiple routes for extracellular reduction in LAB besides FLEET that remain to be elucidated.

Only co-cultures containing *L. plantarum* and *L. lactis* TIL46 or *L. plantarum* and *L. mesenteroides* resulted in accelerated environmental acidification. The pH change was correlated with quinone biosynthesis because the reduction in culture pH was not observed during *L. plantarum* incubation with TIL999. Both co-cultures reached similar pH values during early exponential growth (at 6 h in co-culture with *L. lactis* and 8 h in co-culture with *L. mesenteroides*), suggesting that *L. plantarum* EET metabolism is most active in early exponential-phase growth. Although the results here are from a single time point, they are consistent between the *L. lactis* TIL46 and *L. plantarum* and *L. mesenteroides* strains tested and in agreement with our prior observations showing that incubating *L. plantarum* in kale juice under EET-conducive conditions with a polarized anode resulted in a rapid, transient acidification of the juice concurrent with an increase in metabolic flux and lactic acid production (13). Additional studies will be needed to identify the specific timing and conditions when EET and quinone cross-feeding occurs in food fermentations.

Additionally, the co-culture with *L. mesenteroides* resulted in an initial increase in *L. plantarum* cell numbers. Transient increases in *L. plantarum* growth were similarly observed in kale juice under EET-conducive relative to non-conductive conditions (13). Although the lack of change in *L. plantarum* cell numbers during incubation with either the *L. lactis* TIL46 or TIL999 indicates that quinone-independent factors are likely influencing competition between *L. plantarum* and *L. lactis,* other studies have shown positive outcomes for quinone cross-feeding. Group B *Streptococcus* shifted from fermentation to respiration and exhibited improved growth and survival when grown together with quinone-producing strains of *L. lactis* (56) or *E. faecalis* (3). DHNA, MK-4, and menadione were found to stimulate the growth of strictly anaerobic bacteria isolated from the human intestine (57). Moreover, DHNA produced by *Propionibacterium* increased the growth of *Bifidobacterium*, a bacterial genus that uses fermentation energy conservation metabolism (58). Therefore, these findings suggest that quinone cross-feeding may be pervasive in food and intestinal habitats and that quinones are nutrients important for determining population dynamics in ecosystems wherein fermentation metabolism is prevalent.

### Conclusions

In summary, our results show how quinones affect a microorganism that mainly relies on fermentation energy metabolism and lacks the capacity for quinone biosynthesis but can use exogenous quinones for EET. These findings are important for the understanding microbial interactions in habitats such as fermented foods and animal digestive tracts. Many LAB are particularly well adapted for those habitats, and because those sites also tend to be nutrient-rich, LAB have undergone reductive genome evolution and correspondingly rely on exogenous nutrients (amino acids, nucleosides, etc.) for growth (59). The capacity of *L. plantarum* to use environmental quinones for EET is consistent with its adaptation to those nutrient-rich environments and may afford an increased competitive fitness compared to microorganisms that make those compounds *de novo*. The elucidation of the intracellular and extracellular quinone species needed for EET and the signals used by *L. plantarum* to induce the EET pathway will be valuable to apply these pathways to improve food fermentations and other biotechnological applications.

## MATERIALS AND METHODS

### Strains and culture conditions

All strains and plasmids used in this study are listed in Table 2. Standard laboratory culture medium was used for routine growth of bacteria as follows: *Lactiplantibacillus plantarum* and *Leuconostoc mesenteroides*, De Man-Rogosa-Sharpe medium (MRS) (BD, Franklin Lakes, NJ, USA); *Lactococcus lactis*, M17 (BD) with 2% (wt/vol) glucose (GM17); and *Escherichia coli*, LB (Teknova, Hollister, CA, USA). *L. plantarum* FLEET deletion mutants of *ndh2* or *pplA* were constructed through double-crossover homologous recombination as previously described (13). Strains were grown at 30°C or 37°C when indicated. In place of standard laboratory culture medium, strains were grown (when indicated) in modified MRS (60) with no beef extract and with either 110 mM glucose (gMRS) or 110 mM mannitol (mMRS) as the primary carbon source, or a chemically defined minimal medium (13) with 125 mM glucose (gCDM) or 125 mM mannitol (mCDM) as the primary carbon source.

**TABLE 2:** Strains and plasmids used in this study

Species	Strain	Description	Reference
*L. plantarum*	NCIMB8826R	Rifampicin resistant variant of NCIMB8826	(61)
*L. plantarum*	MLES100	Deletion mutant of NCIMB8826 lacking *ndh2*	(13)
*L. plantarum*	MLES101	Deletion mutant of NCIMB8826 lacking *pplA*	(13)
*L. lactis* subsp. *cremoris*	TIL46	Strain NCDO763 cured of its 2 kb plasmid	(62)
*L. lactis* subsp. *cremoris*	TIL999	Deletion mutant of TIL46 lacking *menC*	(63)
*L. mesenteroides* subsp. *mesenteroides*	ATCC8293	Fermented olives	(59)

### *L. plantarum* growth rate experiments

After overnight growth in MRS at 37°C, *L. plantarum* cells were collected via centrifugation at 10,000 × *g* for 3 min and washed twice in phosphate-buffered saline (PBS) at pH 7.2 (64). Cells were resuspended in PBS at an optical density at 600 nm (OD_600_) of 1 before inoculation into 96-well culture plates (Thermo Fisher Scientific, Waltham, MA) at a final OD_600_ of 0.01 in mMRS. When indicated, mMRS was supplemented with ferric ammonium citrate (VWR, Radnor, PA, USA) (1.25 mM) or ammonium citrate tribasic (Alfa Aesar, Haverhill, MA, USA) (1.25 mM). The following quinones were also supplemented; 1,4-benzenediol (hydroquinone) (Sigma-Aldrich, St. Louis, MO, USA), DHNA (Alfa Aesar), 1,4-naphthoquinone (TCI, Tokyo, Japan), 2-methyl-1,4-naphthoquinone (menadione) (Sigma-Aldrich), vitamin K1 (phylloquinone) (TCI), or vitamin K2 in the form of menaquinone-4 (Sigma-Aldrich) or menaquinone-7 (Sigma-Aldrich). ACNQ was provided by E. Mevers and was prepared as previously reported (10). Quinones were supplemented at 2.5, 5, 10, 20, 50, 100, 150, or 200 µg/mL where indicated. The OD_600_ was measured over 48 h in a Synergy two microplate reader (Biotek, Winooski, VT) set to 37°C with no aeration.

In experiments where cell-free supernatant (CFS) was used in place of quinone supplementation, overnight cultures of *L. lactis* (GM17) or *Leuconostoc* spp. (gMRS) were grown for 18 h at 30°C before normalizing the OD_600_ of these cultures with carbon-free M17 or MRS, respectively. Cells were then centrifuged at 10,000 × *g* for 3 min, and the supernatant was sterile filtered through a 0.22-µm syringe filter. Uninoculated, carbon-free media were also sterile-filtered as a control. Twice-washed *L. plantarum* cells (described above) were resuspended in PBS at an optical density at 600 nm (OD_600_) of 1 before inoculation into 96-well culture plates at a final OD_600_ of 0.01 in 1:1 CFS (or carbon-free media) to 2× (twice-concentrated) mMRS. The OD_600_ was measured over 48 h as described above.

### RNA-seq library construction and transcriptome analysis

The RNA-seq library was constructed and analyzed as previously described (13). In brief, *L. plantarum* NCIMB8826R was grown in mMRS (in triplicate) with or without supplementation of 20-µg/mL DHNA and 1.25-mM ferric ammonium citrate. Cultures were grown at 37°C to exponential phase (OD_600_ = ~1.0) before collection via centrifugation at 10,000 × *g* for 3 min at 4°C. After decanting, cells were flash frozen in liquid N_2_ and stored at −80°C until RNA extraction with acidic phenol:chloroform:isoamyl alcohol (pH 4.5) as previously described (65). RNA was quantified on a Nanodrop 2000c (ThermoFisher) before two rounds of DNAse digestion using the Turbo DNA-free Kit (Invitrogen, Waltham, MA, USA) following the manufacturer’s protocols. RNA quality was checked using a Bioanalyzer RNA 6000 Nano Kit (Agilent Technologies, Santa Clara, CA, USA) (all RNA integrity number [RIN] values > 9), quantified with the Qubit 2.0 RNA HS Assay (Life Technologies, Carlsbad, CA, USA), and depleted of ribosomal-RNA (rRNA) with the RiboMinus Eukaryote Kit (v.2) using specific probes for prokaryotic rRNA (ThermoFisher). The remaining RNA was then fragmented to approximately 200 bp, converted to cDNA, and given barcode sequences using the NEBnext Ultra-directional RNA Library Kit for Illumina (New England Biolabs, Ipswitch, MA, USA) with NEBnext Multiplex Oligos for Illumina (Primer Set 1) (New England Biolabs) following the manufacturer’s instructions. The barcoded cDNA libraries were pooled and run across two lanes of a HiSeq400 (Illumina, San Diego, CA, USA) on two separate runs for 150-bp paired-end reads (http://dnatech.genomecenter.ucdavis.edu/).

After sequencing and demultiplexing, DNA sequences for all 12 samples were first visualized in FastQC (ver. 0.11.8) (66) followed by read trimming with Trimmomatic (ver. 0.39) (67). Remaining reads were aligned to the *L. plantarum* NCIMB8826R chromosome and plasmids using Bowtie2 (ver. 2.3.5) in the (-sensitive) mode (68), and output “.sam” files were converted to “.bam” files with Samtools (ver. 1.9) (69). Aligned reads which corresponded to NCIMB8826R genes, excluding non-coding sequences (e.g., rRNA, tRNA, and trRNA) were enumerated with FeatureCounts in the [--stranded=reverse] mode (ver. 1.6.4) (70). DESeq2 (71) using the Wald test in the R-studio shiny app DEBrowser (ver 1.14.2) (72) was used to quantify differential gene expression based on culture condition. The significance cutoff for differential expression was set to a false discovery rate-adjusted *P* value of ≤0.05 and a log_2_ (fold change) of ≥0.5. Clusters of orthologous groups were also assigned to genes based the eggNOG (ver. 5.0) database (73).

### Hydrogen peroxide production assay

*L. plantarum* NCIMB8826R was grown in mMRS (in triplicate) with or without supplementation of 20 µg/mL DHNA and 1.25 mM ferric ammonium citrate. Cultures were grown at 37°C to exponential phase (OD_600_ = ~1.0) before collection via centrifugation (10,000 g for 3 min). Uninoculated cultures (in triplicate) were used for abiotic hydrogen peroxide production and were sampled after 5 h which was when *L. plantarum* cultures reached OD_600_ = 1 in exponential phase. Hydrogen peroxide was measured fluorometrically with the Fluorimetric Hydrogen Peroxide Assay Kit (Sigma-Aldrich) following the manufacturer’s instructions.

### Ferrihydrite reduction assays

*L. plantarum* strains were first incubated in mMRS for 18 h at 37°C. When indicated, quinones were supplemented at concentrations ranging from 0.01 to 200 µg/mL, and/or ferric ammonium citrate was supplemented at 1.25 mM. Riboflavin was supplemented at 10 µM when indicated. Cells were collected via centrifugation (10,000 × *g* for 3 min) and washed twice in PBS. The OD_600_ was adjusted to two in PBS containing 2.2 mM ferrihydrite (74, 75), 2 mM ferrozine (Sigma-Aldrich), and 55 mM mannitol. Quinones and/or riboflavin were supplemented at the above concentrations when indicated, and uninoculated controls were used to subtract background ferrihydrite reduction by quinones. After 3 h incubation at 37°C, the cells were collected by centrifugation (10,000 g for 5 min) the supernatant was used to determine iron reduction from absorbance measurements at 562 nm with a Synergy two microplate reader. Absorbance was converted to the concentration of reduced iron(II) using a standard curve containing a 2-fold range of FeSO_4_ (Sigma-Aldrich) (0.25 mM to 0.016 mM) dissolved in 10 mM cysteine-HCL (RPI, Mount Prospect, IL, USA) and supplemented with 2 mM ferrozine.

In experiments where cell-free supernatant (CFS) was used in place of PBS as the assay medium, overnight cultures of *L. lactis* (GM17) or *Leuconostoc* spp. (gCDM) were grown for 18 h at 30°C before normalizing the OD_600_ of these cultures with carbon-free M17 or chemically defined minimal medium (CDM), respectively. Cells were then centrifuged (10,000 × *g* for 3 min) and the supernatant was sterile filtered through a 0.22-µm syringe filter. Uninoculated, carbon-free media was also sterile-filtered as a control. The OD_600_ of PBS-washed *L. plantarum* cells was then adjusted to 2 in the CFS or uninoculated media which was supplemented with ferrihydrite, ferrozine, and mannitol as described above. Ferrihydrite reduction assays with *L. lactis* or *Leuconostoc* CFS were carried out at 37°C for 3 h before measuring reduced iron as described above.

### Bioelectrochemical measurements

The bioreactors consisted of double-chamber electrochemical cells (Adams & Chittenden, Berkeley, CA) with a cation exchange membrane (CMI-7000, Membranes International, Ringwood, NJ) that separated them. We used a 3-electrode configuration consisting of an Ag/AgCl (sat. KCl) reference electrode (BASI), a titanium wire counter electrode, and a working electrode of either 6.35-mm-thick graphite felt working electrode of 4 × 4 cm (Alfa Aesar) with a piece of Ti wire threaded as a current collector and connection to the potentiostat. The bioreactors were sterilized by filling them with ddH2O and autoclaving at 121°C for 30 min. After this, each chamber medium was replaced with 150 mL of filter sterilized CDMs (for the working electrode chamber), and 150 mL of M9 medium (BD) (for the counter electrode chamber). The medium (before filter-sterilization) was supplemented with a final concentration of 10 g/L of mannitol and with 20 µg/mL of DHNA diluted 1:1 in DMSO:ddH2O, where appropriate. The medium in the working electrode chamber was mixed with a magnetic stir bar for the course of the experiment and N_2_ gas was continuously purged in the working electrode chamber to maintain anaerobic conditions. Four bioreactors were prepared which differed in the CDM used in the working electrode chamber: two bioreactors contained mCDM supplemented with 20 µg/mL of DHNA (diluted 1:1 in DMSO:ddH2O), and other two bioreactors contained mCDM with no DHNA. All the experiments were tested under 30°C. After approximately 4 h of bubbling the working electrode chamber with N_2_ gas, the working electrode of each bioreactor was polarized. The applied potential to the working electrodes was of 0.2 V vs the Ag/AgCl (sat. KCl) reference electrode. A Bio-Logic Science Instruments potentiostat model VSP-300 was used for performing electrochemical measurements. Once the current density stabilized overnight, the mCDM +DHNA bioreactors were inoculated to a final OD_600_ of ~0.1–0.15 with the cell suspensions of *L. plantarum* prepared in M9 medium. Cell suspensions were prepared from an overnight, statically grown culture (~16–18 h) of *L. plantarum* cultured in MRS medium at 30°C. After 45 h of operating the bioreactors and observing current density production, we collected cells from each bioreactor by vigorously shaking the bioreactors to detach cells from the electrode and collecting the medium from the bioreactors (~150 mL). Cells were collected from each medium by performing 2 cycles of centrifugation (15,228 × *g*, 7 min) and washing with M9 medium. The resulting cell pellets were suspended in oxygen-free M9 medium and inoculated in the two DHNA-free bioreactors, previously polarized to 0.2 V and left overnight to achieve a stable current density baseline. Cyclic voltammetry analyses were performed at a scan rate of 5 mV/s and in the potential region of −0.7 to 0.4 V vs Ag/AgCl.

### Cellular quinone quantification

To prepare cells for assessments of quinone concentrations, *L. plantarum* was grown in mMRS supplemented with 20 µg/mL DHNA and/or 1.25 mM ferric ammonium citrate (FeAC), *L. lactis* in gM17, and *L. mesenteroides* in gMRS. All strains were incubated for 18 h in their respective culture media at 30°C prior to collection by centrifugation at 10,000 × *g* for 3 min. Cells were washed twice in PBS before flash freezing with liquid N_2_. The cell pellet was lyophilized for 18 h and then transferred to a 40 mL glass vial, and ground with a spatula and extracted with 3.0 mL of 2:1 dichloromethane (DCM)/MeOH for 2 h while rocking on gently on a shaker at room temperature. The organic solvent was filtered using a glass plug containing celite and dried under vacuum. The crude material was then resuspended in 200 µL of 2:1 isopropanol (IPA)/MeOH.

For *L. plantarum* NCIMB8826R incubated in mMRS with DHNA or DHNA and FeAc, data were acquired using an Agilent 6530 LC-q-TOF Mass Spectrometer equipped with an uHPLC system were acquired using a Shimadzu 9030 LC-q-ToF Mass Spectrometer equipped with a Nexera LC40 UPLC system. For quinone detection in subsequent experiments (Table 1), 5 µL of the crude cell material was analyzed on a LCMS. Menaquinone analogs were quantified using the Phenomenex Luna 5 µm C5 100 Å (50 × 4.6 mm) under the following method: hold 100% solvent A for 5 min then quickly gradient to 80% solvent A/20% solvent B over 0.1 min, then gradient to 100% solvent B over 34.9 min with a flow rate of 0.4 mL/min (solvent A: 95% H_2_O/5% MeOH +0.1% FA with 5 mM ammonium acetate; solvent B: 60% IPA/35% MeOH/5% H2O + 0.1% FA with 5-mM ammonium acetate). Integrated extracted ion chromatograms for two ion adducts, [M + H]+ and [M + NH4]+, for each menaquinone analog were summed.

### *L. plantarum* co-culturing experiments

*L. plantarum* NCIMB8826R, *L. lactis* TIL46, *L. lactis* TIL999, and *L. mesenteroides* ATCC8293 were grown overnight in gMRS for 18 h at 30°C. Cells were collected by centrifugation at 10,000 *g* for 3 min and washed twice in PBS. Approximately 10^7^ CFU/mL of each strain was inoculated into 125-mL screw-cap bottles containing gCDM supplemented with 1.25 mM ferric ammonium citrate. *L. lactis* TIL46, *L. lactis* TIL999, and *L. mesenteroides* ATCC8293 were also inoculated at approximately 10^7^ CFU/mL in a one-to-one ratio with *L. plantarum* NCIMB8826R. Each strain or strain combination was grown at 30°C in triplicate. Samples were collected for pH, CFU per milliliter enumeration, and Fe^2+^ reduction at the time of inoculation (t = 0), and then either after 6 and 24 h (*L. plantarum* and *L. lactis*) or 4, 8, and 24 h (*L. plantarum* and *L. mesenteroides*). Species in co-culture were differentiated on mMRS agar based on colony size during incubation at 37°C. Fe^2+^ reduction capacity was determined by adding 2.2 mM ferrozine to the supernatant collected after centrifugation of the gCDM at 10,000 × *g* for 5 min and quantifying iron reduction using a Fe^2+^ standard curve as described above.

## Data Availability

*Lactiplantibacillus plantarum* RNA-seq data are available in the NCBI Sequence Read Archive under BioProject accession no. PRJNA717240. A full DEseq2 analysis of RNA-seq data is available in the Harvard Dataverse: https://doi.org/10.7910/DVN/SAQ5AT

## SUPPLEMENTAL MATERIAL

Supplemental figures mbio.02234-23-s0001.docx Figures S1 to S5.

Table S1 mbio.02234-23-s0002.docx Total number of *L. plantarum* genes differentially expressed in DHNA, FeAC, and DHNA+FeAC containing media.

Table S2 mbio.02234-23-s0003.docx *L. plantarum* NCIMB8826R differentially expressed genes in mMRS with DHNA, FeAC, or DHNA+FeAC.

Table S3 mbio.02234-23-s0004.xlsx Complete transcriptome data set.

ASM does not own the copyrights to Supplemental Material that may be linked to, or accessed through, an article. The authors have granted ASM a non-exclusive, world-wide license to publish the Supplemental Material files. Please contact the corresponding author directly for reuse.
