# Supplementary material for: Lactiplantibacillus plantarum uses ecologically relevant, exogenous quinones for extracellular electron transfer
Source: mBio. 2023 Nov 20;14(6):e02234-23. doi: 10.1128/mbio.02234-23 (PMC10746273; doi:10.1128/mbio.02234-23)
Supplement: Supplemental figures — Figures S1 to S5. [file mbio.02234-23-s0001.docx]

**Supplementary Figures**





**Supplementary Figure S1. EET-conducive quinones inhibit *L. plantarum* growth.** Growth of *L. plantarum* in mMRS supplemented with **(A)** DHNA, **(B)** ACNQ, **(C)** 1,4-naphthoquinone, or **(D)** menadione. The avg + SEM of three biological replicates is shown.


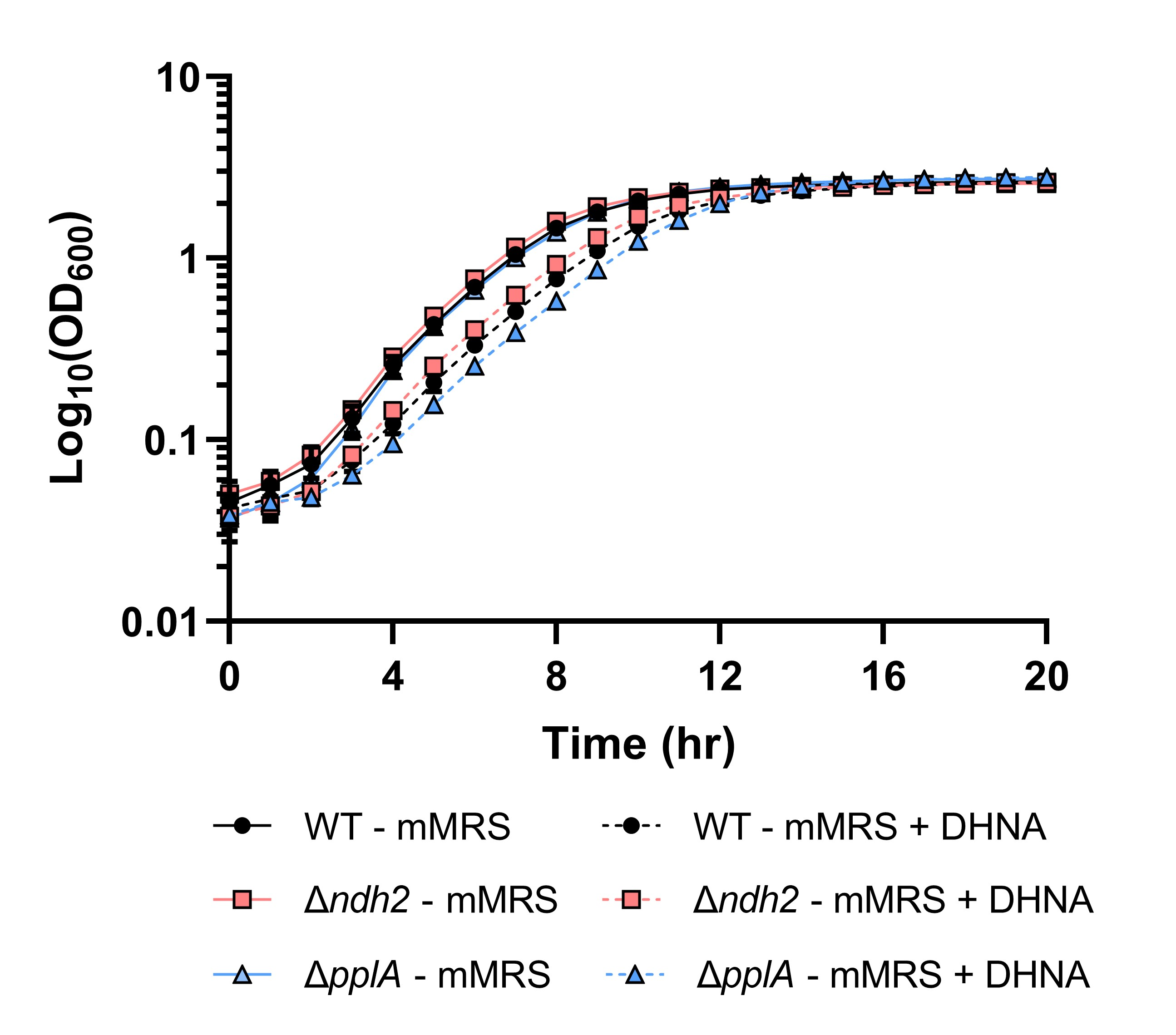


**Supplementary Figure S2. DHNA-induced reduction of *L. plantarum* growth is not influenced by the absence of *ndh2* or *pplA*.** Full growth curve data from **Figure 2A** of wild-type *L. plantarum* NCIMB8826R and the Δ*ndh2* and *ΔpplA* mutants, MLES100 and MLES101, respectively, in mMRS with or without supplementation of 20 μg/mL DHNA. Growth rates were quantified by measuring the change in OD_600nm_ per hour during exponential phase (between 3 to 8 h incubation). OD_600nm_ data was pathlength corrected, which normalizes absorbance based on minor differences in liquid column pathlengths between wells in a 96-well plate.


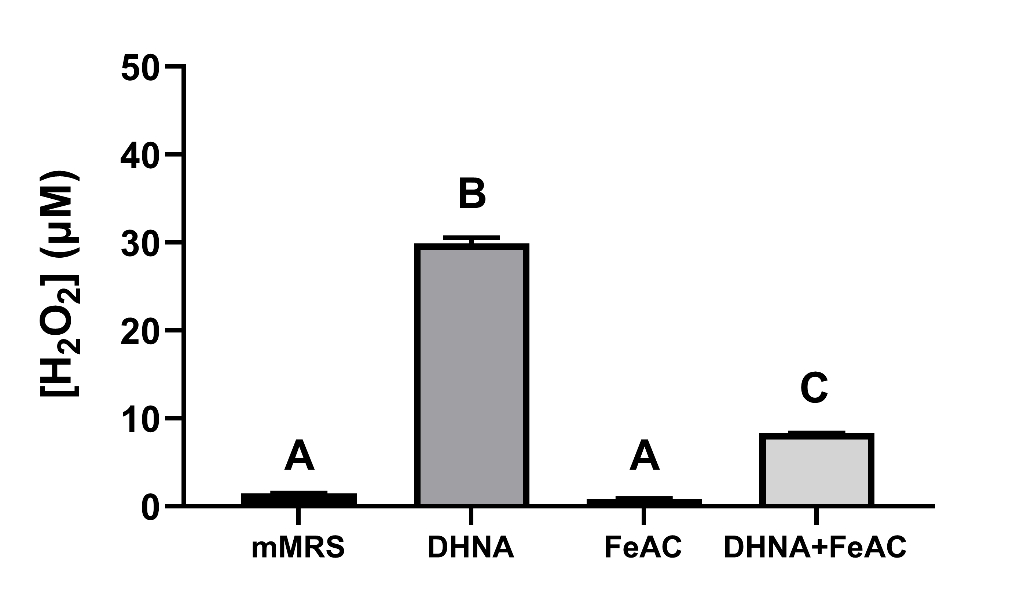


**Supplementary Figure S3. DHNA increases hydrogen peroxide levels in mMRS that are reduced by the presence of FeAC.** Hydrogen peroxide produced in mMRS supplemented with 20 μg/mL DHNA and/or 1.25 mM ferric ammonium citrate (FeAC) after 5 h at 37 °C. *L. plantarum* was inoculated at an OD_600_ = 0.1 and culture supernatant was sampled after 5 h at 37 °C corresponding to when *L. plantarum* reached mid-exponential phase growth. Significant differences in H_2_O_2_ production between culture conditions were determined by one-way ANOVA with Tukey’s post-hoc test. Different letters indicate statistically significant differences between conditions (p < 0.05). The avg + stdev of three biological replicates is shown.


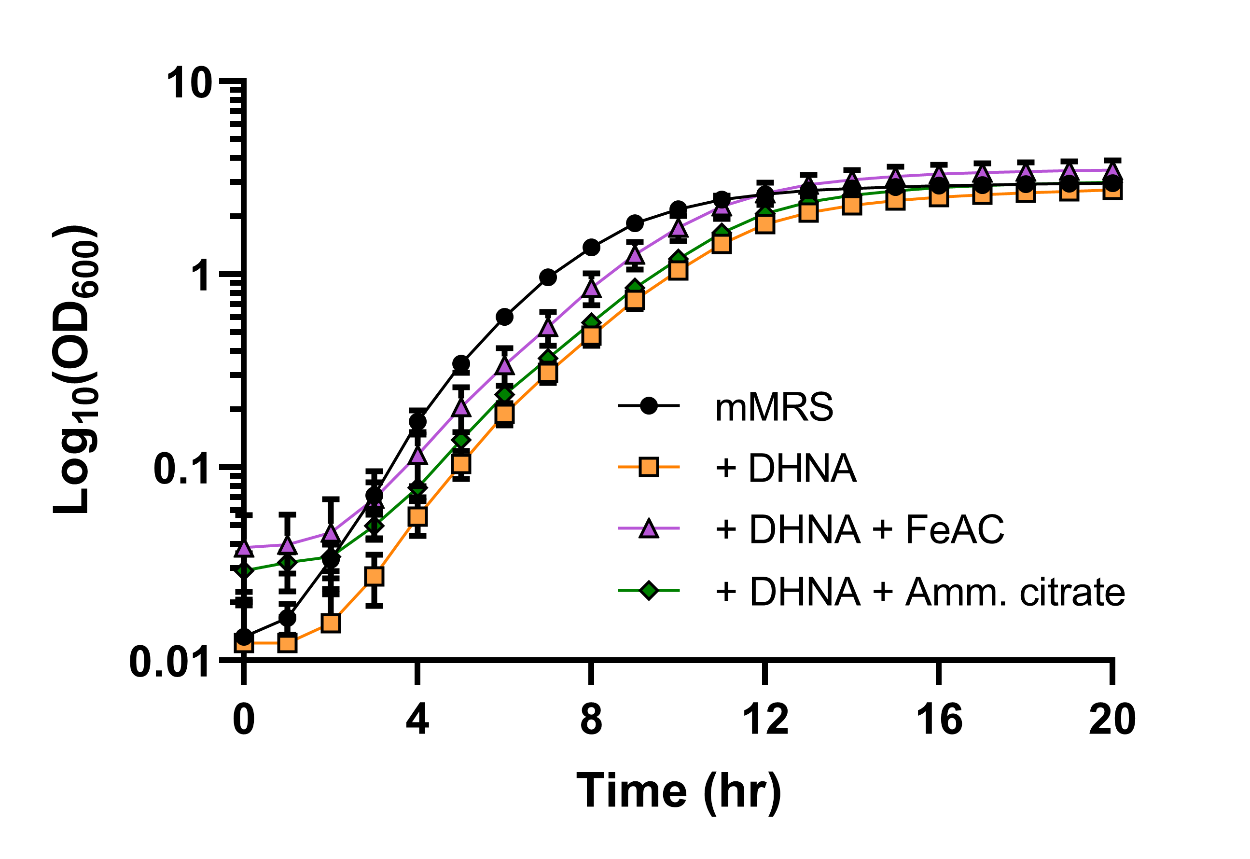


**Supplementary Figure S4.** **DHNA reduces *L. plantarum* growth and this is ameliorated by soluble ferric iron in the laboratory culture medium.** Full growth curve data from **Figure 2B** of *L. plantarum* NCIMB8826R in mMRS with or without supplementation of 20 μg/mL DHNA and 1.25 mM ferric ammonium citrate (FeAC) or 1.25 mM ammonium citrate. Growth rates were quantified by measuring the change in OD_600nm_ per hour during exponential phase. OD_600nm_ data was pathlength corrected, which normalizes absorbance based on minor differences in liquid column pathlengths between wells in a 96-well plate.





**Supplementary Figure S5. FeAC supplementation in mMRS does not increase *L. plantarum* growth rate.** Growth rates were determined by measuring the change in OD_600nm_ per hour during exponential phase. The avg + SEM of three biological replicates is shown. OD_600nm_ data was collected without pathlength correction, which normalizes absorbance based on minor differences in liquid column pathlengths between wells in a 96-well plate.


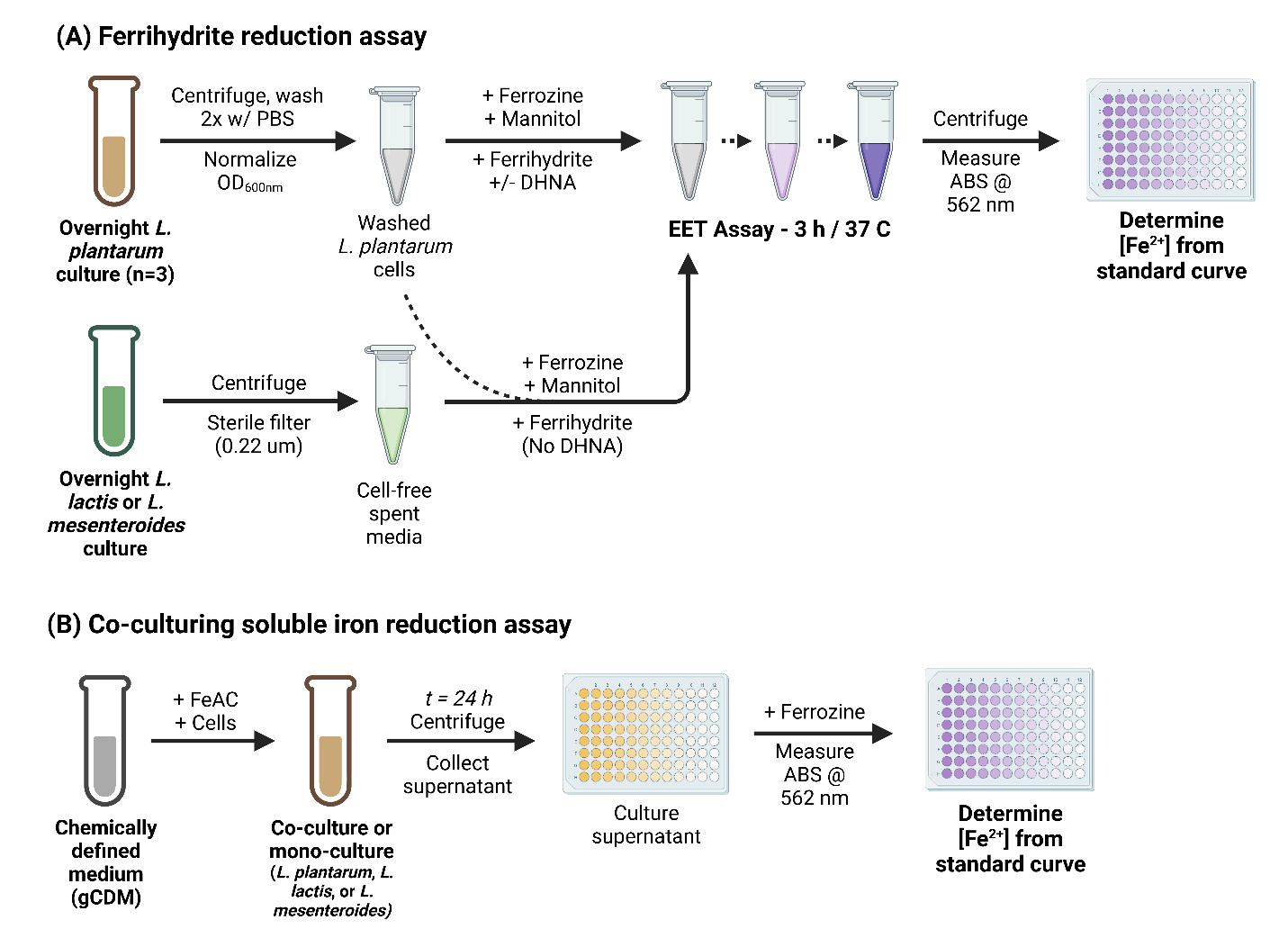


**Supplementary Figure S6. Visualized methods for iron reduction experiments using insoluble or soluble iron.** For **(A)** ferrihydrite (insoluble iron), *L. plantarum* cells are collected and washed in 1x PBS (pH 7.2) before normalizing cell numbers via OD_600nm_. EET is performed after ferrozine, mannitol, ferrihydrite, and (where indicated) DHNA or spent growth media from *L. lactis* or *L. mesenteroides* is added. Supernatant is collected after 3 h and used to determine Fe^2+^ from absorbance at 562_nm_. In co-culturing experiments, **(B)** ferric ammonium citrate (soluble iron, FeAC) is added to gCDM along with *L. plantarum*, *L. lactis*, and/or *L. mesenteroides* cells. After 24 h, supernatant is collected and ferrozine is added to instantly determine Fe^2+^ colorimetrically (absorbance at 562_nm_).


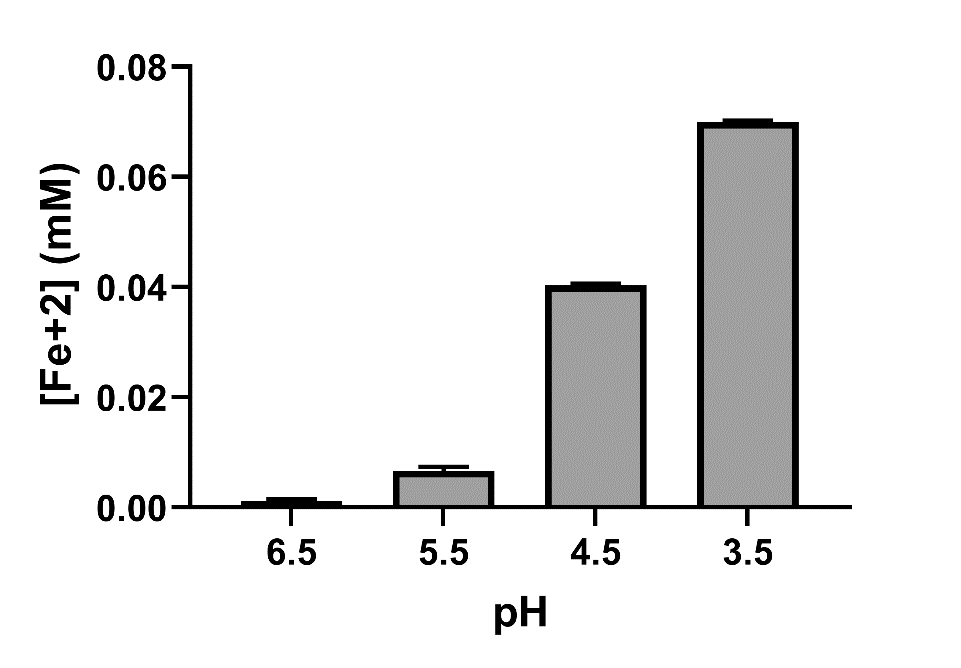


**Supplementary Figure S7. Spontaneous iron reduction in CDM.** pH was reduced with ~4% v/v lactic acid. Iron reduction was detected colorimetrically with 2 mM ferrozine. The avg + stdev of three replicates is shown.
