# Supplementary material for: Lactiplantibacillus plantarum uses ecologically relevant, exogenous quinones for extracellular electron transfer
Source: mBio. 2023 Nov 20;14(6):e02234-23. doi: 10.1128/mbio.02234-23 (PMC10746273; doi:10.1128/mbio.02234-23)
Supplement: Table S1 — Total number of L. plantarum genes differentially expressed in DHNA, FeAC, and DHNA+FeAC containing media. [file mbio.02234-23-s0002.docx]

**Supplementary Table S1. Total number of *L. plantarum* genes differentially expressed in DHNA, FeAC, and DHNA+FeAC containing media.**

| **Growth condition** | **# Upregulated Genes** | **# Downregulated Genes** | **Total # Differentially Expressed Genes** |
| --- | --- | --- | --- |
| DHNA | 452 | 473 | 925 |
| FeAC | 35 | 72 | 107 |
| DHNA+FeAC | 123 | 169 | 292 |

Differential expression of all genes in *L. plantarum* during exponential growth in mMRS supplemented with DHNA (20 μg/mL) and/or ferric ammonium citrate (1.25 mM) compared to mMRS. Differences in gene expression levels were considered significant when there was an FDR-adjusted *p*-value ≤ 0.05 and log_2_ expression fold-change ≥ 0.5.
