# Supplementary material for: Lactiplantibacillus plantarum uses ecologically relevant, exogenous quinones for extracellular electron transfer
Source: mBio. 2023 Nov 20;14(6):e02234-23. doi: 10.1128/mbio.02234-23 (PMC10746273; doi:10.1128/mbio.02234-23)
Supplement: Table S2 — L. plantarum NCIMB8826R differentially expressed genes in mMRS with DHNA, FeAC, or DHNA+FeAC. [file mbio.02234-23-s0003.docx]

**Supplementary Table S2. *L. plantarum* NCIMB8826R differentially expressed genes in mMRS with DHNA, FeAC, or DHNA+FeAC.**

| **Locus Tag** | **Gene** | **Product** | **COG ID#** | **DHNA**  **Log2 FC** | **DHNA**  **p-adj** | **FeAC Log2 FC** | **FeAC**  **p-adj** | **DHNA+**  **FeAC**  **Log2 FC** | **DHNA+**  **FeAC**  **p-adj** |
| --- | --- | --- | --- | --- | --- | --- | --- | --- | --- |
| *Oxidation-reduction* | | | | | | | | | |
| lp_1253 | gshR2 | glutathione reductase | COG1249 | **2.86** | **0.000** | **-1.66** | **0.000** | **-1.95** | **0.000** |
| lp_1822 | gshR3 | glutathione reductase | COG1249 | **1.04** | **0.000** | 0.02 | 0.978 | 0.05 | 0.917 |
| lp_3267 | gshR4 | glutathione reductase | COG1249 | **1.48** | **0.000** | 0.00 | 0.997 | 0.24 | 0.221 |
| lp_3578 | kat | catalase | COG0753 | **1.88** | **0.000** | -0.88 | **0.000** | **-1.30** | **0.000** |
| lp_1939 | lp_1939 | oxidoreductase, medium chain dehydrogenases/reductase (MDR)/zinc-dependent alcohol dehydrogenase-like family | COG0604 | **1.73** | **0.000** | -0.30 | 0.057 | -0.80 | **0.000** |
| lp_1836 | mrsB | protein-methionine-S-oxide reductase | COG0229 | **2.32** | **0.000** | **-1.13** | **0.000** | **-1.49** | **0.000** |
| lp_1835 | msrA2 | protein-methionine-S-oxide reductase | COG0225 | **2.42** | **0.000** | **-1.16** | **0.000** | **-1.38** | **0.000** |
| lp_3449 | nox5 | NADH oxidase | COG0446 | 0.69 | **0.000** | **-2.40** | **0.000** | **-2.34** | **0.000** |
| lp_2544 | npr2 | NADH peroxidase | COG0446 | **2.93** | **0.000** | -0.79 | **0.000** | 0.15 | 0.516 |
| lp_0694 | nrdH | glutaredoxin-like protein nrdH | COG0695 | -0.05 | 0.711 | 0.14 | 0.515 | **-1.05** | **0.000** |
| lp_2788 | panE | 2-dehydropantoate 2-reductase | COG1893 | **1.23** | **0.000** | -0.60 | 1.000 | 0.84 | 1.000 |
| lp_2629 | pox3 | pyruvate oxidase | COG0028 | **1.70** | **0.000** | **-2.31** | **0.000** | **-2.96** | **0.000** |
| lp_3589 | pox5 | pyruvate oxidase | COG0028 | 0.44 | **0.001** | **-1.18** | **0.000** | **-2.42** | **0.000** |
| lp_2323 | tpx | thiol peroxidase | COG2077 | **1.20** | **0.000** | 0.15 | 0.289 | 0.14 | 0.578 |
| lp_2270 | trxA2 | thioredoxin | COG3118 | 0.53 | **0.000** | -0.02 | 0.938 | -0.49 | **0.004** |
| lp_0761 | trxB | thioredoxin reductase (NADPH) | COG0492 | 0.78 | **0.000** | -0.19 | 0.075 | -0.48 | **0.000** |
|  |  |  |  |  |  |  |  |  |  |
| *Amino acid transport and metabolism* | | | | | | | | | |
| lp_1084 | aroD1 | shikimate 5-dehydrogenase | COG0169 | **2.53** | **0.000** | -0.31 | 0.751 | **1.08** | 1.000 |
| lp_2037 | aroF | chorismate synthase | COG0082 | **2.21** | **0.000** | -0.39 | 0.609 | 0.63 | 1.000 |
| lp_0861 | bcaP | branched-chain amino acid permease | COG0531 | **2.12** | **0.000** | 0.16 | 0.357 | -0.08 | 0.603 |
| lp_0981 | brnE | branched-chain amino acid transport protein | COG4392 | **2.88** | **0.000** | 0.29 | 0.608 | 0.21 | 1.000 |
| lp_0982 | brnF | branched-chain amino acid transport protein | COG1296 | **2.26** | **0.000** | 0.01 | 0.992 | 0.15 | 0.830 |
| lp_1245 | hicD2 | L-2-hydroxyisocaproate dehydrogenase | COG0039 | **3.20** | **0.000** | 0.17 | 0.561 | 0.40 | 0.062 |
| lp_1297 | lp_1297 | S-methylmethionine transport protein | COG0833 | **2.55** | **0.000** | -0.17 | 0.715 | 0.14 | 0.823 |
| lp_2920 | lp_2920 | proline-containing amino acid or peptide transport protein | COG0531 | **3.23** | **0.000** | -0.26 | 0.705 | 0.56 | 0.546 |
| lp_3214 | lp_3214 | cystathionine ABC transporter, substrate binding protein | COG0834 | **2.14** | **0.000** | -0.01 | 0.986 | 0.10 | 0.808 |
| lp_1375 | metE | homocysteine S-methyltransferase (cobalamin-independent) | COG0620 | **2.16** | **0.000** | -0.47 | 0.529 | 0.88 | 0.420 |
| lp_1298 | metH | homocysteine S-methyltransferase (cobalamin-dependent) | COG2040 | **2.35** | **0.000** | -0.16 | 0.792 | 0.41 | 0.515 |
| lp_2536 | oahS | O-acetylhomoserine sulfhydrylase | COG2873 | **2.20** | **0.000** | -0.56 | 0.395 | 0.91 | 0.462 |
| lp_1261 | oppA | oligopeptide ABC transporter, substrate binding protein | COG4166 | **6.15** | **0.000** | 0.15 | 0.424 | 0.09 | 0.657 |
| lp_1262 | oppB | oligopeptide ABC transporter, permease protein | COG0601 | **5.00** | **0.000** | 0.11 | 0.696 | 0.17 | 0.486 |
| lp_1263 | oppC | oligopeptide ABC transporter, permease protein | COG1173 | **4.79** | **0.000** | 0.06 | 0.832 | 0.15 | 0.508 |
| lp_1264 | oppD | oligopeptide ABC transporter, ATP-binding protein | COG0444 | **4.79** | **0.000** | 0.02 | 0.960 | 0.04 | 0.869 |
| lp_1265 | oppF | oligopeptide ABC transporter, ATP-binding protein | COG4608 | **4.58** | **0.000** | 0.15 | 0.438 | -0.02 | 0.911 |
| lp_2708 | pucR | purine transport regulator | COG2508 | **3.56** | **0.000** | -0.17 | 0.836 | 0.58 | 0.483 |
| lp_0203 | serA | D-3-phosphoglycerate dehydrogenase | COG0111 | **6.41** | **0.000** | -0.20 | 0.581 | -0.34 | 0.457 |
| lp_0204 | serC | phosphoserine aminotransferase | COG1932 | **6.04** | **0.000** | -0.18 | 0.691 | -0.11 | 0.850 |
| lp_2308 | thrA2 | aspartate kinase | COG0527 | **2.43** | **0.000** | 0.29 | 0.058 | 0.15 | 0.498 |
|  |  |  |  |  |  |  |  |  |  |
| *Cell membrane biosynthesis* | | | | | | | | | |
| lp_1680 | accA2 | acetyl-CoA carboxylase, carboxyl transferase subunit alpha | COG0825 | **1.10** | **0.000** | 0.73 | **0.000** | **3.85** | **0.000** |
| lp_1676 | accB2 | acetyl-CoA carboxylase, biotin carboxyl carrier protein | COG0511 | **1.25** | **0.000** | 0.90 | **0.000** | **3.92** | **0.000** |
| lp_1678 | accC2 | acetyl-CoA carboxylase, biotin carboxylase subunit | COG0439 | **1.23** | **0.000** | 0.87 | **0.000** | **3.92** | **0.000** |
| lp_1679 | accD2 | acetyl-CoA carboxylase, carboxyl transferase subunit beta | COG0777 | **1.26** | **0.000** | 0.88 | **0.000** | **3.98** | **0.000** |
| lp_1672 | acpA2 | acyl carrier protein | COG0236 | **1.33** | **0.000** | **1.11** | **0.000** | **3.90** | **0.000** |
| lp_1673 | fabD | [acyl-carrier protein] S-malonyltransferase | COG0331 | **1.35** | **0.000** | **1.05** | **0.000** | **4.00** | **0.000** |
| lp_1675 | fabF | 3-oxoacyl-[acyl-carrier protein] synthase II | COG0304 | **1.12** | **0.000** | 0.89 | **0.000** | **3.68** | **0.000** |
| lp_1674 | fabG1 | 3-oxoacyl-[acyl-carrier protein] reductase | COG1028 | **1.19** | **0.000** | 0.97 | **0.000** | **3.80** | **0.000** |
| lp_1671 | fabH2 | 3-oxoacyl-[acyl-carrier protein] synthase III | COG0332 | **1.37** | **0.000** | **1.19** | **0.000** | **3.97** | **0.000** |
| lp_1681 | fabI | enoyl-[acyl-carrier protein] reductase (NADH) | COG0623 | **1.06** | **0.000** | 0.79 | **0.000** | **3.79** | **0.000** |
| lp_1670 | fabZ1 | (3R)-hydroxyacyl-[acyl carrier protein] dehydratase | COG0764 | **1.31** | **0.000** | **1.29** | **0.000** | **3.92** | **0.000** |
| lp_1677 | fabZ2 | (3R)-hydroxymyristoyl-[acyl carrier protein] dehydratase | COG0764 | **1.39** | **0.000** | 0.96 | **0.000** | **4.08** | **0.000** |
|  |  |  |  |  |  |  |  |  |  |
| *Respiration* | | | | | | | | | |
| lp_1491 | mobA | molybdopterin-guanine dinucleotide biosynthesis protein MobA | COG0746 | 0.12 | 0.829 | -0.01 | 1.000 | 1.00 | 1.000 |
| lp_1492 | moaC | molybdopterin precursor synthase MoaC | COG0315 | 0.75 | 0.171 | 0.11 | 1.000 | 1.78 | 1.000 |
| lp_1493 | mobB | molybdopterin-guanine dinucleotide biosynthesis protein MobB | COG1763 | 0.75 | 0.165 | 0.39 | 1.000 | 1.56 | 1.000 |
| lp_1494 | moeA | molybdopterin biosynthesis protein MoeA | COG0303 | 0.34 | 0.250 | 0.17 | 0.846 | **1.55** | **0.001** |
| lp_1495 | moaB | molybdopterin biosynthesis protein MoaB | COG0521 | 0.76 | 0.140 | 0.45 | 1.000 | 1.60 | 1.000 |
| lp_1496 | moeB | molybdopterin biosynthesis protein MoeB | COG0476 | 0.74 | 0.014 | 0.16 | 0.880 | **1.50** | **0.001** |
| lp_1497 | narG | nitrate reductase, alpha chain | COG5013 | -0.04 | 0.778 | 0.14 | 0.508 | **1.32** | **0.000** |
| lp_1498 | narH | nitrate reductase, beta chain | COG1140 | -0.17 | 0.317 | 0.14 | 0.691 | **1.08** | **0.000** |
| lp_1499 | narJ | nitrate reductase, delta chain | COG2180 | -0.32 | 0.140 | -0.04 | 0.963 | 0.98 | 0.000 |
| lp_1500 | narI | nitrate reductase, gamma chain | COG2181 | -0.36 | 0.047 | 0.05 | 0.938 | 0.95 | 0.000 |
| lp_1125 | cydA | cytochrome D ubiquinol oxidase, subunit I | COG1271 | -0.11 | 0.209 | 0.26 | 0.001 | -0.10 | 0.598 |
| lp_1126 | cydB | cytochrome D ubiquinol oxidase, subunit II | COG1294 | -0.03 | 0.729 | 0.28 | 0.001 | -0.01 | 0.948 |
| lp_1128 | cydC | cytochrome D ABC transporter, ATP-binding and permease protein | COG4988 | 0.16 | 0.106 | 0.19 | 0.219 | 0.03 | 0.865 |
| lp_1129 | cydD | cytochrome D ABC transporter, ATP-binding and permease protein | COG4987 | 0.18 | 0.040 | 0.13 | 0.425 | -0.06 | 0.733 |
|  |  |  |  |  |  |  |  |  |  |
| *FLEET locus* | | | | | | | | | |
| lp_1066 | dmkB | heptaprenyl diphosphate synthase component II | COG0142 | **-0.50** | **0.000** | 0.06 | 0.799 | 0.18 | 0.426 |
| lp_1067 | eetB | heptaprenyl diphosphate synthase component I | COG4769 | -0.40 | 0.000 | 0.12 | 0.562 | 0.39 | 0.072 |
| lp_1068 | eetA | extracellular protein, DUF1312 family | COG5341 | -0.42 | 0.000 | 0.04 | 0.890 | 0.38 | 0.090 |
| lp_1069 | ndh2 | NADH dehydrogenase, membrane-anchored | COG1252 | -0.24 | 0.005 | 0.06 | 0.719 | **0.63** | **0.000** |
| lp_1070 | pplA | lipoprotein precursor, FMN-binding protein | COG4939 | **-0.53** | **0.000** | -0.03 | 0.870 | **0.67** | **0.000** |
| lp_1072 | fmnB | thiamin biosynthesis lipoprotein ApbE | COG1477 | **-0.52** | **0.000** | 0.03 | 0.895 | 0.32 | 0.102 |
| lp_1073 | ATPase_2 | ABC transporter, ATP-binding protein | COG1122 | -0.24 | 0.005 | 0.06 | 0.740 | 0.15 | 0.513 |
| lp_1074 | ATPase_1 | ABC transporter, ATP-binding protein | COG1122 | -0.24 | 0.005 | 0.03 | 0.897 | -0.01 | 0.967 |
| lp_1075 | fmnA | ABC transporter, permease protein | COG0619 | 0.05 | 0.638 | 0.18 | 0.188 | 0.19 | 0.447 |
|  |  |  |  |  |  |  |  |  |  |
| *Menaquinone biosynthesis* | | | | | | | | | |
| lp_1135 | lp_1135 | 1,4-dihydroxy-2-naphthoate octaprenyltransferase | COG1575 | 0.23 | 0.012 | 0.13 | 0.446 | 0.17 | 0.494 |
| lp_1715 | lp_1715 | 1,4-dihydroxy-2-naphthoate octaprenyltransferase, UbiA prenyltransferase family | COG1575 | -0.09 | 0.417 | -0.02 | 0.952 | -0.38 | 0.003 |
| lp_1546 | dmkA | 1,4-dihydroxy-2-naphthoate octaprenyltransferase, UbiA family | COG1575 | -0.34 | 0.001 | 0.09 | 0.649 | 0.22 | 0.246 |
| lp_3431 | ubiE | menaquinone/ubiquinone biosynthesis methyltransferase | COG2226 | 0.31 | 0.031 | -0.07 | 0.886 | 0.19 | 0.505 |

^a^ Log2 fold-change (FC) differences in *L. plantarum* gene expression levels in mMRS supplemented with DHNA (20 μg/mL) and/or ferric ammonium citrate (1.25 mM) compared to mMRS. Bold values indicate a log_2_ expression fold-change > 0.5 and an FDR-adjusted *p*-value < 0.05.
